# Genomic convergence of mobilome-associated multidrug resistance and host-adaptation determinants in a postoperative *Raoultella ornithinolytica* isolate carrying *bla*_KPC-2_, *bla*_NDM-5,_ and a truncated *mcr-10*-like element

**DOI:** 10.3389/fcimb.2026.1966531

**Published:** 2026-09-25

**Authors:** Ziheng Wang, Guangping Xu, Ruodan Li, Jiaxu Qin, Ruiming Bao, Zihao Chen, Jie Li, Peng Zhang

**Affiliations:** 1 Department of Laboratory Medicine, The First Affiliated Hospital of Wannan Medical University, Wuhu, China; 2 Department of Transfusion Medicine, The First Affiliated Hospital of Wannan Medical University, Wuhu, China

**Keywords:** *bla*_KPC-2_, *bla*_NDM-5_, conjugative plasmid, *mcr-10*-like, One Health, *Raoultella ornithinolytica*

## Abstract

**Objective:**

To characterize the convergence of resistance determinants, plasmid-mediated dissemination, and host adaptation in the postoperative *Raoultella ornithinolytica* isolate RO0725 through integrated genomic analyses and phenotypic validation. We further sought to evaluate its clinical and One Health risks.

**Methods:**

Long-read sequencing was used to generate a complete genome. Subsequent analyses assessed phylogeny, average nucleotide identity, resistance genes, and plasmid comparisons. Phenotypic validation included conjugation assays, bile salt tolerance, biofilm formation, and a *Galleria mellonella* infection model.

**Results:**

The RO0725 genome comprised one chromosome and five circular plasmids. RO0725 carried 14 antimicrobial-resistance genes distributed across the chromosome and three resistance-associated plasmids. Chromosomal determinants included intrinsic resistance-associated genes such as *bla*_ORN-1_, *oqxA10*, *oqxB9*, and *fosA9*. The plasmid pRO0725–1 carried a truncated *mcr* - 10-like element, whereas pRO0725–2 harbored *bla*_KPC-2_, *bla*_CTX-M-3_, *bla*_TEM-1_, *qnrS1*, *dfrA14*, *arr-3*, and *aac(6′)-Ib-AKT*. The IncX3-like plasmid pRO0725–3 carried *bla*_NDM-5_ and *ble*-MBL. Both pRO0725–2 and pRO0725–3 exhibited transferable potential in conjugation assays. RO0725 did not form a near-clonal cluster (defined as ≤10 core-genome SNPs) with any public isolates. RO0725 contained host-adaptation-associated determinants involved in adhesion (*ecp*, *fim*, and *mrk*), iron acquisition (*ent*, *fep*, *ybt*, *irp*, *fyuA*, and *iutA*), stress response (*phoP*, *rcsB*, *rpoS*, *acrAB*, and *ompA*), and cellulose biosynthesis (*bcs*). Phenotypically, RO0725 exhibited enhanced bile salt tolerance, weak biofilm formation, and moderate lethality in the *G. mellonella* model.

**Conclusion:**

RO0725 represents a convergence of transferable multidrug-resistance plasmids and host-adaptation determinants. These findings indicate that *R. ornithinolytica* may act as both an opportunistic pathogen and a potential reservoir for the interspecies dissemination of resistance genes. They support its inclusion in hospital and One Health antimicrobial-resistance surveillance.

## 1 Introduction

Carbapenemases and plasmid-mediated resistance to polymyxins and tigecycline are progressively limiting treatment options for infections caused by Enterobacterales (Song et al., 2026). Resistance determinants such as *bla*_KPC_, *bla*_NDM_, *mcr*, and *tmexCD-toprJ* often occur on plasmids, integrons, and transposon-associated regions. These mobile locations facilitate their horizontal dissemination across bacterial species and ecological niches (Wang CZ. et al., 2021; Jia et al., 2025; Ye et al., 2025). In recent years, *bla*_KPC_, *bla*_NDM_, *mcr-10*, *tmexCD-toprJ*, and *tet(X)* have been identified in *Raoultella ornithinolytica*. These findings suggest that this species may serve as an important reservoir and transmission vehicle for multidrug-resistance determinants (Cortés-Ortíz et al., 2021; Wang M. et al., 2021; Quintela-Baluja et al., 2022; Iyer and Iken, 2023; Sun X. et al., 2023; Karpenko et al., 2024).

The genus *Raoultella*, formerly classified within the genus *Klebsiella*, currently includes several recognized species, such as *R. ornithinolytica*, *R. planticola*, and *R. terrigena* (Appel et al., 2021). Among these species, *R. ornithinolytica* has received particular clinical attention because it is the species most frequently reported in human infections and has demonstrated a greater association with multidrug-resistant clinical isolates (Wang M. et al., 2021). *R. ornithinolytica* is an encapsulated, facultatively anaerobic, Gram-negative bacillus widely distributed in aquatic environments, soil, food, animals, and healthcare-associated settings. Because of its phenotypic similarity to *Klebsiella oxytoca*, conventional methods may misidentify it. Matrix-assisted laser desorption/ionization time-of-flight mass spectrometry (MALDI-TOF MS) and whole-genome sequencing have improved its clinical recognition (Etani et al., 2023). This organism has been associated with bloodstream, urinary tract, pulmonary, biliary tract, soft-tissue, and postoperative infections. Such infections occur particularly in immunocompromised patients and those undergoing invasive procedures (Prada-Avella et al., 2021; Goodman et al., 2022; Hinchcliffe et al., 2022; Suito et al., 2022; De Coster et al., 2024; Huang et al., 2024; Sun et al., 2024). However, rapidly progressive invasive infections have also occurred in otherwise healthy individuals, indicating that its pathogenic potential is not restricted to traditionally susceptible populations (Baek et al., 2023).

From a One Health perspective, isolates from clinical cases, companion animals, river sediments, and aquaculture wastewater carry antimicrobial-resistance genes and mobile plasmids. These findings suggest that *R. ornithinolytica* may contribute to the circulation of resistance determinants among humans, animals, and the environment (Quintela-Baluja et al., 2022; Alam et al., 2023; Jia et al., 2025). Here, we performed complete genomic and phenotypic characterization of RO0725, an isolate recovered from postoperative drainage fluid. The strain co-harbored *bla*_KPC-2_, *bla*_NDM-5_, and a truncated *mcr-10*-like element. We conducted phylogenetic and plasmid structural analyses, conjugation assays, and host-adaptation analyses to evaluate its dissemination potential and clinical significance.

## 2 Materials and methods

### 2.1 Clinical information, bacterial identification, and antimicrobial susceptibility testing

A 59-year-old man with Stanford type A aortic dissection and coronary artery disease underwent ascending aortic resection, prosthetic graft replacement, and coronary artery bypass grafting on January 6, 2026. An *R. ornithinolytica* isolate was recovered from postoperative drainage fluid. It was identified using MALDI-TOF MS (VITEK MS system, bioMérieux), confirmed by whole-genome sequencing, and designated RO0725. MICs were determined using the VITEK 2 Compact system (bioMérieux). Ceftazidime–avibactam and aztreonam–avibactam were tested by disk diffusion. Results were interpreted according to CLSI M100, 36th edition. Aztreonam–avibactam results were interpreted according to EUCAST v16.1.

### 2.2 Whole-genome sequencing, assembly, and genome annotation

Genomic DNA from RO0725 was extracted using the NucleoBond^®^ HMW DNA Kit (Macherey-Nagel, Germany). DNA quality was assessed using NanoDrop, Qubit 4.0, and agarose gel electrophoresis. Libraries were sequenced on the Oxford Nanopore PromethION platform using R10.4.1 flow cells. After contamination assessment with Kraken2 v2.1.3, Oxford Nanopore reads were assembled *de novo* using Canu v2.2 with the following parameters: genomeSize=5.5m, correctedErrorRate=0.105, and minReadLength=1000. The resulting assembly was polished using Medaka v2.0.1 with the r1041_e82_400bps_sup_g610 model. Genome circularization was confirmed by terminal-overlap analysis (Koren et al., 2017; Di Pilato et al., 2025). Sequencing depth was calculated using minimap2 v2.24-r1122 and SAMtools v1.18. The final assembly comprised one complete chromosome and five circular plasmids (Li et al., 2009; Li, 2021).

Genome annotation was performed using Bakta v1.10.1 and included CDSs, tRNAs, rRNAs, and ncRNAs. Functional annotation was then performed against the COG and KEGG databases (**Supplementary Figures S1 and S2**) (Kanehisa et al., 2017; Schwengers et al., 2021; Galperin et al., 2025). CRISPR-Cas systems and prophages were predicted using CRISPRCasFinder v4.2.20 and PhiSpy v4.2.30, respectively (Akhter et al., 2012; Couvin et al., 2018). Antimicrobial-resistance genes, virulence factors, and plasmid replicons were screened using ABRicate v1.4.0. The CARD v4.0.1, ResFinder v4.7.2, VFDB, and PlasmidFinder v3.0.3 databases were used (Carattoli and Hasman, 2020; Florensa et al., 2022; Liu et al., 2022; Alcock et al., 2023). Identity and coverage thresholds were set at ≥80%. Plasmid mobility was assessed using MOB-suite v3.1.9, oriTfinder, and MacSyFinder v2.1.6 with MacSyLib v1.0.4, CONJScan v2.0.2, and HMMER v3.2.1 (Abby et al., 2014; Li et al., 2018; Robertson and Nash, 2018). Sequence comparisons were performed using NCBI BLAST+ v2.17.0+ (Camacho et al., 2009).

### 2.3 Global genomic dataset construction and phylogenomic analysis

Public *R. ornithinolytica* genomes were retrieved using NCBI Datasets CLI v18.33.1. After deduplication and quality control, we selected the 100 genomes most closely related to RO0725 in the preliminary global core-genome SNP analysis. Together with RO0725, these genomes yielded a dataset of 101 genomes. Coding sequences were uniformly predicted using Pyrodigal v3.7.1 (Larralde, 2022). Core genes were identified using NCBI BLAST+ v2.17.0+, with RO0725 as the reference. Thresholds were ≥95% nucleotide identity, ≥90% alignment coverage, and presence in ≥99% of genomes. Maximum-likelihood phylogenetic analysis was performed using IQ-TREE v3.1.3. ModelFinder was used to evaluate eight candidate nucleotide substitution models, including combinations of GTR/HKY with Gamma and FreeRate heterogeneity models. The best-fit model was selected according to the Bayesian information criterion (BIC), resulting in GTR+F+R4 as the optimal model. Because the phylogenetic reconstruction was based on core-genome SNP alignment, ascertainment bias correction was applied using the ASC option. The final tree was inferred with 1,000 ultrafast bootstrap replicates and 1,000 SH-aLRT replicates (Trifinopoulos et al., 2016). Whole-genome ANI was calculated using pyfastani v0.6.1, and all results were visualized using ChiPlot (Xie et al., 2023; Larralde et al., 2025).

### 2.4 Comparative analysis of antimicrobial resistance and mobilome features

Presence–absence matrices were generated from the annotation results for antimicrobial-resistance genes, virulence genes, and plasmid replicons. The matrices were visualized using ChiPlot (Xie et al., 2023). Complete circular reference plasmids homologous to pRO0725-1, pRO0725-2, and pRO0725–3 were screened and aligned using NCBI BLAST+ v2.17.0+ (Camacho et al., 2009). After deduplication and sequence-orientation normalization, circular plasmid comparisons were generated using gbdraw (Kawato, 2026). The truncated *mcr-10*-like element, *dfrA14* integron, multidrug-resistance region, and *bla*_NDM-5_ region were compared using blastn. The results were visualized as linear synteny maps.

### 2.5 Characterization of host-adaptation determinants and phenotypic validation

Biofilm formation by RO0725 and the reference strains was assessed using a crystal-violet assay in 96-well plates. Six replicate wells were used per strain, and results were classified according to Stepanović et al. (2007). Bile salt tolerance was evaluated by comparing viable counts before and after exposure to 50 mg/mL bile salts for 4 h. Virulence was assessed using a *Galleria mellonella* infection model. Larvae were injected with different bacterial inocula through the last left proleg. Each group contained 10 larvae. Hypervirulent *Klebsiella pneumoniae* NTUH-K2044 and PBS served as positive and negative controls, respectively. Survival was recorded at regular intervals and analyzed using Kaplan–Meier curves.

Because sodium azide is subject to regulatory restrictions in China and was unavailable, plasmid conjugation assays used RO0725 as the donor. A laboratory-preserved *K. pneumoniae* strain, KP033, served as the recipient. KP033 was resistant to doxycycline but susceptible to polymyxin, aztreonam, and carbapenems. Log-phase donor and recipient cultures were mixed at a 1:2 volume ratio and statically co-incubated overnight at 37 °C. Putative pRO0725–1 transconjugants were selected using 16 μg/mL doxycycline and 16 μg/mL polymyxin. Putative pRO0725–2 and pRO0725–3 transconjugants were selected using doxycycline plus aztreonam or ceftazidime–avibactam, respectively. Aztreonam was used at 64 μg/mL, and ceftazidime–avibactam was used at 16/4 μg/mL. Candidate colonies were purified and identified using the VITEK MS system (bioMérieux, France). Transfer of the corresponding plasmids was confirmed by PCR targeting the truncated *mcr-10*-like element, *bla*_KPC-2_, and *bla*_NDM-5_, respectively (**Supplementary Table S1**). The conjugative transfer frequency was calculated as the number of confirmed transconjugant colony-forming units (CFUs) divided by the number of donor cells (CFUs) used in the mating assay and expressed as transconjugants per donor cell.

### 2.6 Statistical analysis

Data are presented as the mean ± standard deviation. Differences among groups were evaluated using one-way analysis of variance followed by Tukey’s multiple-comparison test. Survival curves were compared using the log-rank (Mantel–Cox) test.

## 3 Results

### 3.1 Clinical course and antimicrobial susceptibility

A 59-year-old man with Stanford type A aortic dissection and coronary artery disease underwent ascending aortic resection, prosthetic graft replacement, and coronary artery bypass grafting on January 6, 2026. He received multiple antimicrobial agents during hospitalization, and *R. ornithinolytica* RO0725 was isolated from postoperative drainage fluid on January 21 (**Supplementary Figure S3**).

RO0725 was resistant to most β-lactams, carbapenems, fluoroquinolones, tobramycin, and colistin. It was intermediate to amikacin and norfloxacin and susceptible to tetracyclines, tigecycline, and trimethoprim–sulfamethoxazole. The resistance phenotype was largely consistent with the presence of *bla*_KPC-2_, *bla*_NDM-5_, *qnrS1*, and *aac(6′)-Ib*. However, detection of *dfrA14* was discordant with susceptibility to trimethoprim–sulfamethoxazole (**Table 1**).

**Table 1:** Antimicrobial susceptibility profile and corresponding resistance determinants of *R. ornithinolytica* strain RO0725.

Antimicrobial category	Antimicrobial agent	MIC (µg/mL)	Interpretation	Corresponding resistance determinant(s)
Monobactam/β-lactamase inhibitor combination	Aztreonam–avibactam	ND^a^	R	*bla*_KPC-2_; *bla*_NDM-5_; *bla*_CTX-M-3_; *bla*_TEM-1_; *bla*_ORN-1_
β-Lactam/β-lactamase inhibitor combinations	Amoxicillin–clavulanate	≥32	R	*bla*_KPC-2_; *bla*_NDM-5_; *bla*_TEM-1_; *bla*_ORN-1_
	Ticarcillin–clavulanate	≥128	R	
	Piperacillin–tazobactam	≥128	R	
Cephalosporin/β-lactamase inhibitor combinations	Cefoperazone–sulbactam	≥64	R	*bla*_KPC-2_; *bla*_NDM-5_; *bla*_CTX-M-3_; *bla*_ORN-1_
	Ceftazidime–avibactam	ND^a^	R	
Penicillins	Ticarcillin	≥128	R	*bla*_KPC-2_; *bla*_NDM-5_; *bla*_TEM-1_; *bla*_ORN-1_
	Piperacillin	≥128	R	
Cephamycins	Cefotetan	≥64	R	*bla*_KPC-2_; *bla*_NDM-5_; *bla*_ORN-1_
Monobactams	Aztreonam	≥64	R	*bla*_KPC-2_; *bla*_CTX-M-3_; *bla*_TEM-1_; *bla*_ORN-1_
Cephalosporins	Cefazolin	≥64	R	*bla*_KPC-2_; *bla*_NDM-5_; *bla*_CTX-M-3_; *bla*_TEM-1_; *bla*_ORN-1_
	Cefuroxime	≥64	R	
	Cefuroxime axetil	≥64	R	
	Ceftazidime	≥64	R	
	Ceftriaxone	≥64	R	
	Cefpodoxime	≥8	R	
	Cefotaxime	≥64	R	
	Cefepime	≥32	R	
Carbapenems	Imipenem	≥16	R	*bla*_KPC-2_; *bla*_NDM-5_;
	Doripenem	≥8	R	
	Meropenem	≥16	R	
Aminoglycosides	Amikacin	8	I	*aac(6′)-Ib*
	Tobramycin	8	R	
Fluoroquinolones	Levofloxacin	≥8	R	*qnrS1*
	Ciprofloxacin	≥4	R	
Quinolones	Nalidixic acid	≥32	R	
Fluoroquinolones	Moxifloxacin	≥8	R	
	Norfloxacin	8	I	
Tetracyclines	Tetracycline	2	S	
	Doxycycline	2	S	
	Minocycline	2	S	
Glycylcyclines	Tigecycline	≤0.5	S	
Folate pathway antagonists	Trimethoprim–sulfamethoxazole	40	S	*dfrA14*
Polymyxins	Colistin	≥16	R	Truncated *mcr-10*-like element

^a^Ceftazidime–avibactam and aztreonam–avibactam were tested by Kirby–Bauer disk diffusion; MICs were not determined. Therefore, only categorical interpretations are reported. MIC results and the ceftazidime–avibactam result were interpreted using CLSI M100, 36th edition (2026). The aztreonam–avibactam result was interpreted using EUCAST Clinical Breakpoint Tables v16.1 (2026). All interpretations followed *Enterobacterales* criteria. For agents without applicable CLSI breakpoints, the original laboratory interpretation was retained. Resistance determinants were assigned from the RO0725 whole-genome sequence. Abbreviations: MIC, minimum inhibitory concentration; ND, not determined; S, susceptible; I, intermediate; R, resistant.

### 3.2 Genomic features and functional annotation

Nanopore sequencing generated 63,256 high-quality reads totaling 999.42 Mb, with a read N50 of 25,523 bp and a mean quality score of 22.7. *De novo* assembly yielded six complete circular replicons totaling 5,878,252 bp, with an overall GC content of 55.54%. These replicons comprised one chromosome and five plasmids (**Table 2A**). The chromosome was 5,526,141 bp long and had a GC content of 55.77%. pRO0725–1 to pRO0725–5 were 165,530, 85,621, 50,555, 46,434, and 3,971 bp, respectively (**Supplementary Figure S4**). We annotated 5,480 CDSs, 89 tRNAs, 25 rRNAs, 81 ncRNAs, and one tmRNA and predicted five prophage regions. Genome annotation was initially performed using Bakta v1.10.1, covering CDS, tRNA, rRNA, and ncRNA. As the final genome record was processed via the NCBI Prokaryotic Genome Annotation Pipeline (PGAP), the resulting annotation statistics may differ slightly from those reported in the manuscript. Five candidate CRISPR arrays were detected, but no associated *cas* genes were identified.

**Table 2:** Genomic and mobilization characteristics of the chromosome and plasmids of *R. ornithinolytica* RO0725.

A. Basic genomic characteristics							
Replicon	Length (bp)	GC content (%)	Sequencing depth (×)	Relative copy number	No. of CDSs	Incompatibility group	Major genetic cargo
Chromosome	5,526,141	55.77	160.62	1	5,098	NA	*bla*_ORN-1_, *oqxA10*, *oqxB9*, *fosA9*;
pRO0725-1	165,530	52.64	259.04	1.61	161	IncFIB(K)/IncFII-like	Truncated *mcr-10*-like element; *bcs*, *ars* and *ter*
pRO0725-2	85,621	53.21	236.53	1.47	94	IncN/IncU	*bla_KPC-2_*, *bla*_CTX-M-3_, *bla*_TEM-1_, *qnrS1*, *dfrA14*, *arr-3* and *aac(6′)-Ib-AKT*
pRO0725-3	50,555	46.57	71.86	0.45	58	IncX3-like^a^	*bla*_NDM-5_ and *ble-*MBL
pRO0725-4	46,434	53.47	45.31	0.28	63	ND	–
pRO0725-5	3,971	43.49	105.45	0.66	6	Col(pHAD28)	–

B. Conjugative-transfer characteristics							
Plasmid	oriT	Relaxase	T4CP	T4SS/MPF system		Predicted mobility	
pRO0725-1	+	+	+	MPF_F/T4SS_typeF		Conjugative	
pRO0725-2	+	+	+	MPF_T/T4SS_typeT		Conjugative	
pRO0725-3	+	+	+	MPF_T/T4SS_typeT		Conjugative	
pRO0725-4	ND	ND	ND	ND		ND	
pRO0725-5	ND	ND	ND	ND		ND	

^a^pRO0725–3 was designated IncX3-like based on its highly conserved IncX3 backbone, although it did not meet the 80% identity and 80% coverage threshold for a formal PlasmidFinder IncX3 call. ND, not determined because no corresponding mobility-associated elements (including oriT, relaxase, type IV coupling protein, or type IV secretion system) were identified; NA, not applicable.

### 3.3 Phylogenomic position and genomic relatedness

A phylogenetic tree of 101 isolates was constructed from 79,563 SNPs across 4,346 core genes and resolved six major clades. RO0725 clustered within Clade IV. Its closest relative was the environmental isolate NUITM-VR1 (GCF_019973635.1), recovered in Vietnam in 2021. The next closest relatives were the human clinical isolate 0 (GCF_905219255.2), recovered in Spain in 2016, and two leafy-vegetable isolates from the United Kingdom. The latter were KB18LG-0270–2 and KB18LG-0270-1, recovered in 2019 (**Figure 1**).

**Figure 1:**
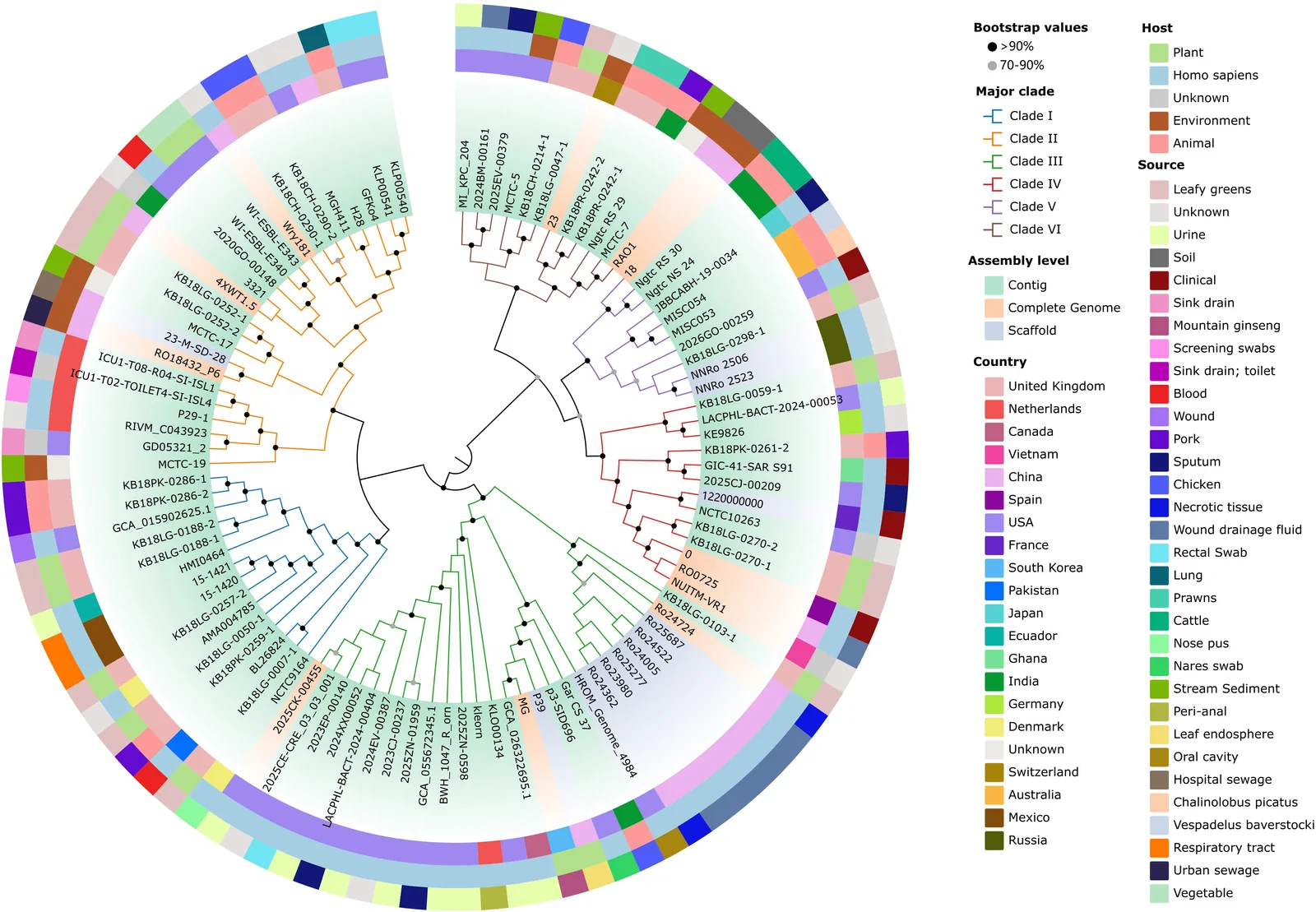
Phylogenetic relationships among *R. ornithinolytica* isolates. Branch colors indicate major clades, and tip colors indicate assembly levels. The three annotation rings, from inner to outer, represent country, host, and isolation source. Circles at the nodes indicate bootstrap support.

Pairwise ANI values among the 101 isolates ranged from 98.79% to 99.99%. Values between RO0725 and the other 100 isolates ranged from 99.15% to 99.88%. RO0725 had the highest ANI with GCF_905219255.2 (99.88%), followed by KB18LG-0270-2 (99.80%), KB18LG-0270-1 (99.77%), and NUITM-VR1 (99.77%) (**Supplementary Figure S5**). Minimum-spanning-tree analysis showed that RO0725 differed from NUITM-VR1 and GCF_905219255.2 by 124 and 139 core-genome SNPs, respectively. RO0725 did not form a near-clonal cluster, defined by ≤10 SNPs, with any other isolate (**Supplementary Figure S6**).

### 3.4 Distribution of resistance genes, virulence genes, and plasmid replicons

The clinical isolate RO0725 carried 14 antimicrobial-resistance genes, including *bla*_CTX-M-3_, *bla*_KPC-2_, *bla*_NDM-5_, *bla*_ORN-1_, *bla*_TEM-1_, *aac(6′)-Ib-AKT*, *dfrA14*, *oqxA10*, *oqxB9*, *qnrS1*, *fosA9*, *arr-3*, *ble-MBL*, and a truncated *mcr* - 10-like element. These acquired ARGs were consistently identified by both CARD and ResFinder (**Supplementary Figure S7**). The detected *mcr-10*-like sequence had 100% nucleotide identity across the aligned region but covered only 68.52% of the full-length reference sequence. It was therefore classified as truncated. In contrast, NUITM-VR1 (GCF_019973635.1) carried a truncated *mcr-10*-like element (**Figure 2**). The corresponding isolate was collected in Vietnam in 2021. These resistance determinants were distributed across the chromosome and three resistance-associated plasmids. Chromosome-encoded determinants mainly included intrinsic resistance-associated genes such as *bla*_ORN-1_, *oqxA10*, *oqxB9*, and *fosA9*. The plasmid pRO0725–1 carried the truncated *mcr* - 10-like element, whereas pRO0725–2 harbored *bla*_KPC-2_ together with additional acquired resistance genes, including *bla*_CTX-M-3_, *bla*_TEM-1_, *aac(6′)-Ib-AKT*, *qnrS1*, *dfrA14*, and *arr-3.* The plasmid pRO0725–3 carried *bla*_NDM-5_ and *ble-MBL.*

**Figure 2:**
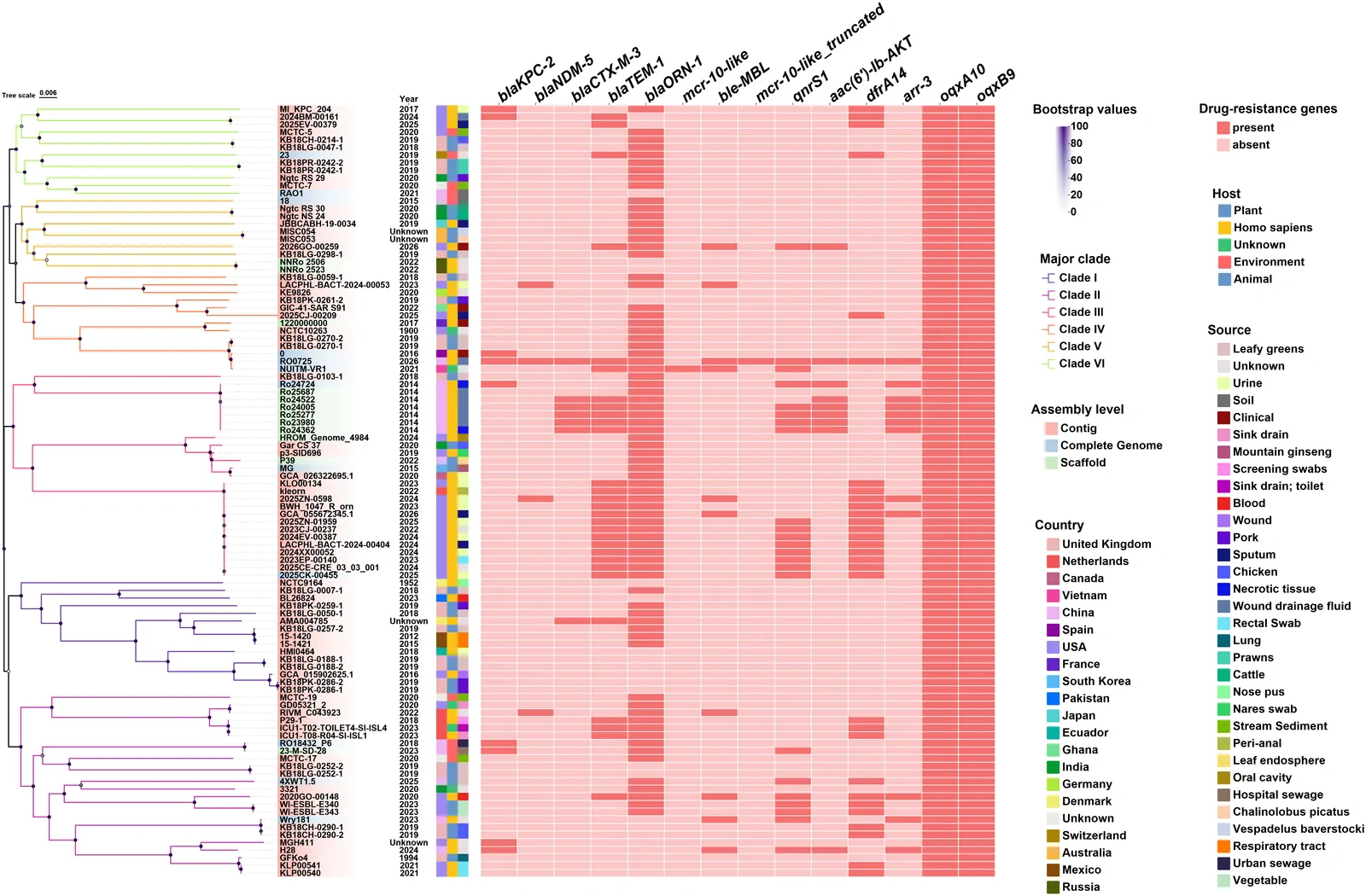
Phylogenetic distribution of selected antimicrobial-resistance genes among 101 *R. ornithinolytica* isolates. The heatmap indicates the presence or absence of 14 representative antimicrobial-resistance genes, including clinically relevant acquired resistance determinants. The maximum-likelihood phylogenetic tree was constructed using IQ-TREE v3.1.3 based on core-genome SNP alignment with the GTR+F+R4 model, selected by ModelFinder, with ascertainment bias correction and 1,000 ultrafast bootstrap and SH-aLRT replicates. The tree and associated heatmap were visualized using ChiPlot. Metadata tracks indicate year, country, host, specimen source, and assembly level. Branch colors represent major clades.

In the comparative genomic analysis of 101 isolates, we identified 85 antimicrobial-resistance genes. *oqxA10* and *oqxB9* were present in all isolates, whereas *fosA*- and *bla*_ORN_-family genes were also widely distributed (**Figure 2**). In contrast, acquired β-lactamases and other resistance determinants were detected in a subset of isolates with variable distribution patterns. *bla*_KPC-2_ and *bla*_NDM-5_ were detected in nine and four isolates, respectively. RO0725 was the only isolate co-harboring *bla*_KPC-2_, *bla*_NDM-5_, and an *mcr* - 10-like element.

Using the VFDB-based systematic screening approach, we identified 56 virulence-associated genes among the 101 *R. ornithinolytica* isolates. Of these, 35 were conserved across all isolates, whereas the remaining genes showed variable distributions among strains. These genes were mainly associated with fimbrial adhesion, iron acquisition, stress regulation, and outer-membrane functions. They indicated a relatively conserved virulence-associated genetic backbone. RO0725 carried 40 virulence-associated genes. These included the fimbrial genes *ecpA*, *fim*, and *mrk* and the iron-acquisition genes *ent*, *fep*, *ybt*, *irp*, *fyuA*, and *iutA*. Several variable type VI secretion system-associated genes and *east1* were absent (**Supplementary Figure S9**).

We detected 41 plasmid replicon types. IncFIB(K), IncFII(pKP91), and Col(pHAD28) were the most prevalent and occurred in 31, 24, and 22 isolates, respectively (**Supplementary Figure S8**). RO0725 carried five replicon types: Col(pHAD28), IncFIB(K), IncFII(pKP91), IncN, and IncU. This combination was not observed in any other isolate and further highlighted the complexity of its plasmid repertoire (**Figure 3**).

**Figure 3:**
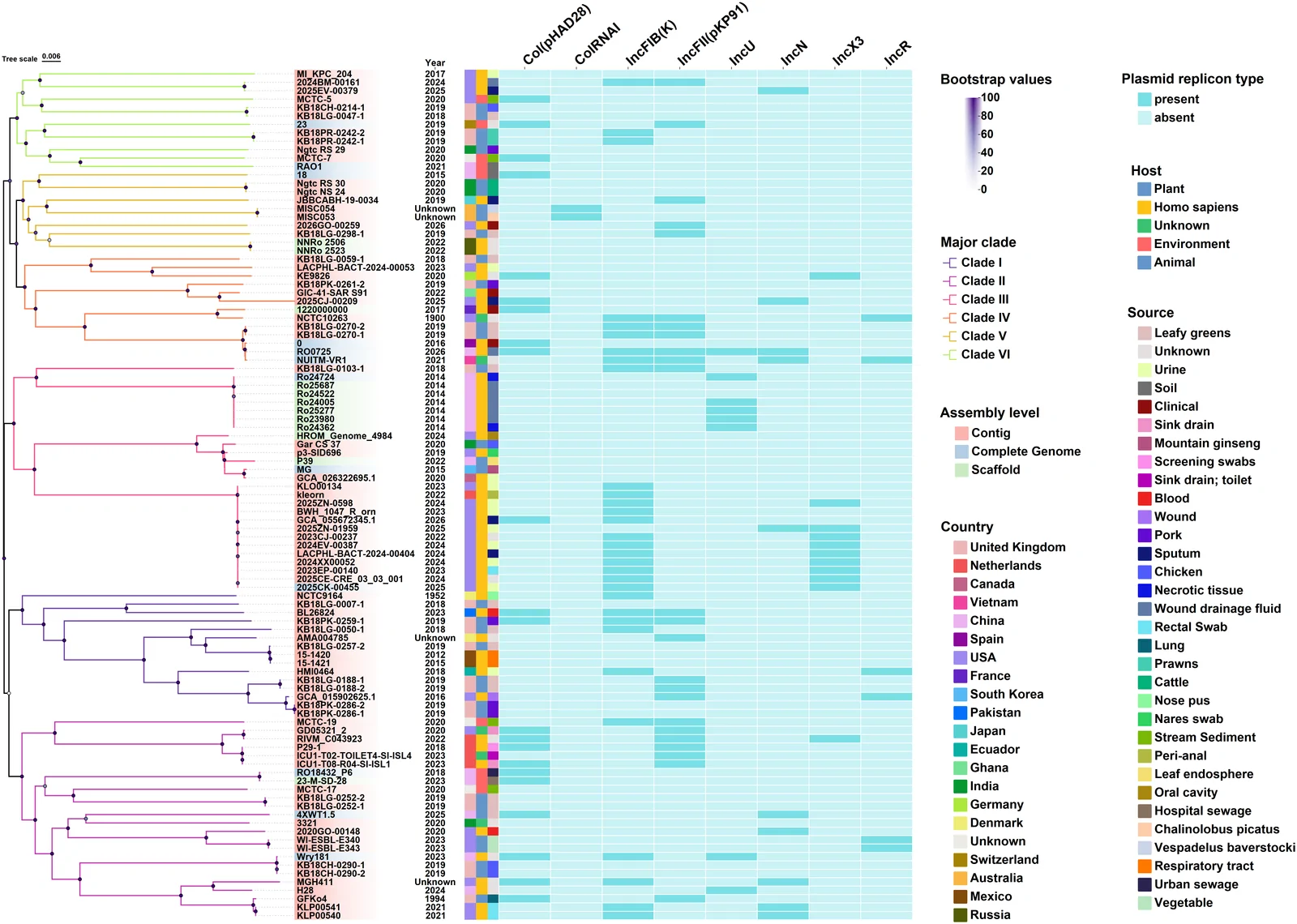
Phylogenetic distribution of selected plasmid replicon types among 101 *R. ornithinolytica* isolates. The heatmap indicates the presence or absence of selected plasmid replicon types detected among the study isolates. The maximum-likelihood phylogenetic tree was constructed using IQ-TREE v3.1.3 based on core-genome SNP alignment with the GTR+F+R4 model, selected by ModelFinder, with ascertainment bias correction and 1,000 ultrafast bootstrap and SH-aLRT replicates. The tree and heatmap were visualized using ChiPlot. Metadata tracks indicate year, country, host, specimen source, and assembly level. Branch colors represent major clades.

### 3.5 Comparative analysis of resistance plasmids

pRO0725–1 was a 165,530-bp IncFIB(K)-positive F-type large plasmid. Its overall backbone was most similar to CP193782.1, with 93.29% query coverage and 98.78% nucleotide identity. AP025011.1, PQ136552.1, and MN175502.1 retained only partially homologous backbones. Replication, maintenance, and conjugative-transfer regions were relatively conserved. In contrast, regions associated with cellulose biosynthesis, arsenic and tellurite tolerance, and the genomic context of the truncated *mcr-10*-like element showed marked structural variation (**Figure 4**). In addition to resistance-associated elements, pRO0725–1 carried a 13.4-kb cellulose biosynthesis-associated locus containing *bcsO*, *bcsQ*, *bcsA*, *bcsB*, *bcsG*, *bcsF*, and BcsC/BcsD/BcsZ-like genes. The *bcsG* and *bcsF* genes showed opposite orientation relative to the *bcsOQABCDZ*-related region. An IS1N-associated element and an IS6-family transposase were identified upstream and downstream of this locus, respectively, suggesting possible involvement of mobile elements in its acquisition. oriTfinder, MOB-suite, and CONJScan consistently predicted an oriT, a relaxase, a type IV coupling protein, and a complete F-type type IV secretion system. Nevertheless, no pRO0725-1-positive transconjugants were recovered under the experimental conditions used (**Table 2B**).

**Figure 4:**
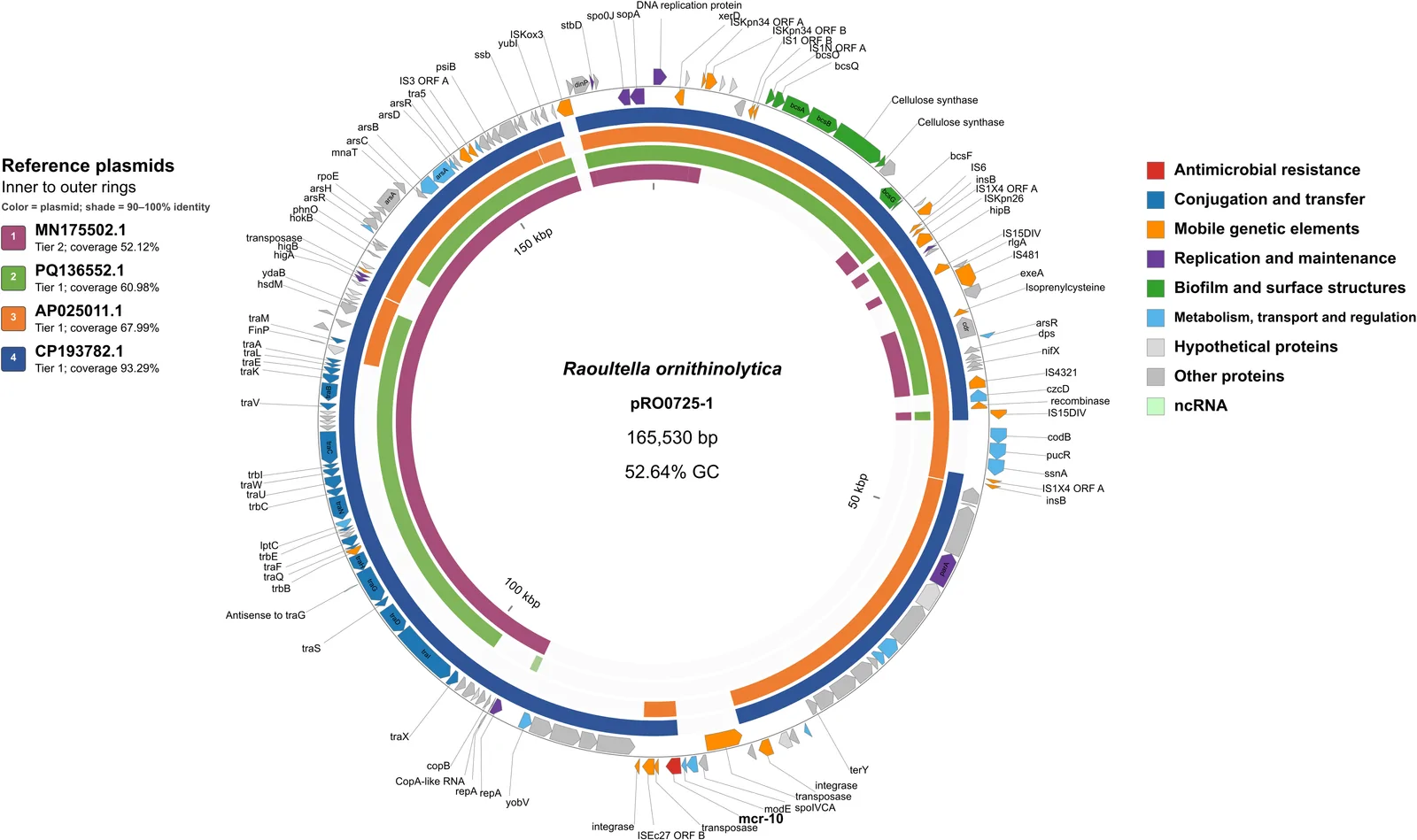
Circular comparative map of pRO0725–1 and four reference plasmids. Colored inner rings indicate regions with 90–100% nucleotide identity. Outer arrows indicate genes classified by function, including the *mcr-10*-like element.

pRO0725–2 was an 85,621-bp IncN/IncU multidrug-resistance plasmid (**Table 2B**). It shared approximately 72.5%–81.8% of its backbone with reference plasmids from diverse Enterobacterales, with nucleotide identities exceeding 95%. The hosts included *Enterobacter hormaechei*, *K. pneumoniae*, *Citrobacter koseri*, *E. coli*, and *Morganella morganii*. Replication, stable-maintenance, and conjugative-transfer modules were relatively conserved. In contrast, the resistance region contained integrons, insertion sequences, and transposases and showed extensive insertions, deletions, and rearrangements. The plasmid carried *bla*_KPC-2_, *bla*_CTX-M-3_, *bla*_TEM-1_, *qnrS1*, *dfrA14*, *arr-3*, and *aac(6′)-Ib-AKT* (**Figure 5**). In conjugation assays, pRO0725–2 transferred to KP033 at a mean frequency of (6.3 ± 0.43) × 10^-^³. This result was consistent with its complete oriT–relaxase–T4CP–T4SS architecture.

**Figure 5:**
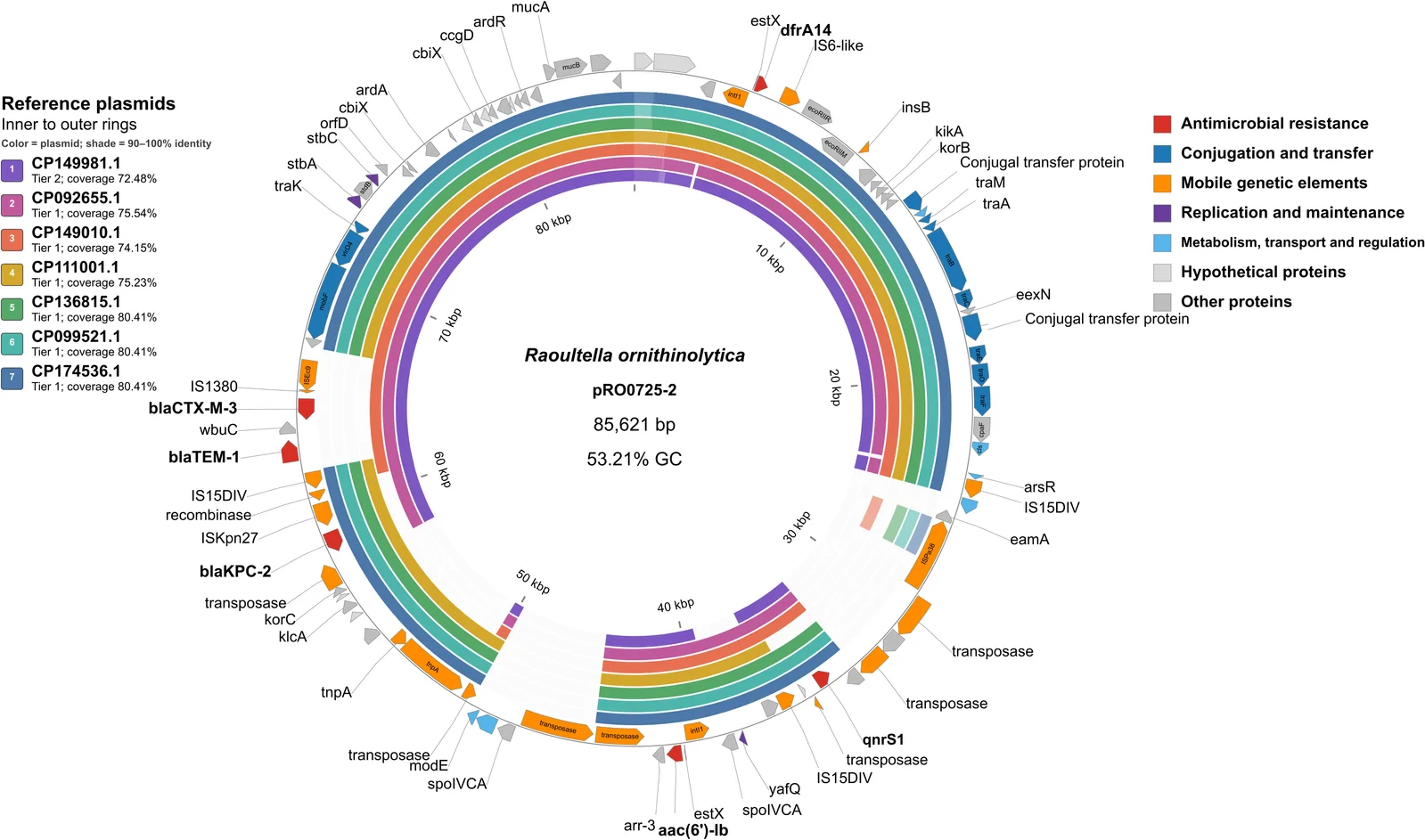
Circular comparative map of pRO0725–2 and seven reference plasmids. Colored inner rings indicate regions with 90–100% nucleotide identity, and outer arrows represent functionally classified genes, including *bla*_KPC-2_.

pRO0725–3 was a 50,555-bp IncX3-like resistance plasmid carrying the *bla*_NDM-5_–*ble*-MBL resistance module (**Table 2B**). It showed 100% query coverage and 99.97% nucleotide identity to CP094514.1. It also shared 86.36% of a highly conserved backbone with KX023261.1 and LC521848.1. These findings place it within the widely disseminated IncX3-like *bla*_NDM-5_–*ble*-MBL plasmid family. Structural differences were mainly confined to local insertion sequences and expanded accessory regions. Replication, partitioning, and conjugative-transfer modules remained highly conserved (**Figure 6**). pRO0725–3 transferred to KP033 at a mean frequency of (3.5 ± 2.1) × 10^-4^. This frequency was approximately 18-fold lower than that of pRO0725-2. Although its predicted T4SS completeness was lower than that of the other two plasmids, the conjugation assay confirmed its capacity for horizontal transfer (**Supplementary Figure S10**).

**Figure 6:**
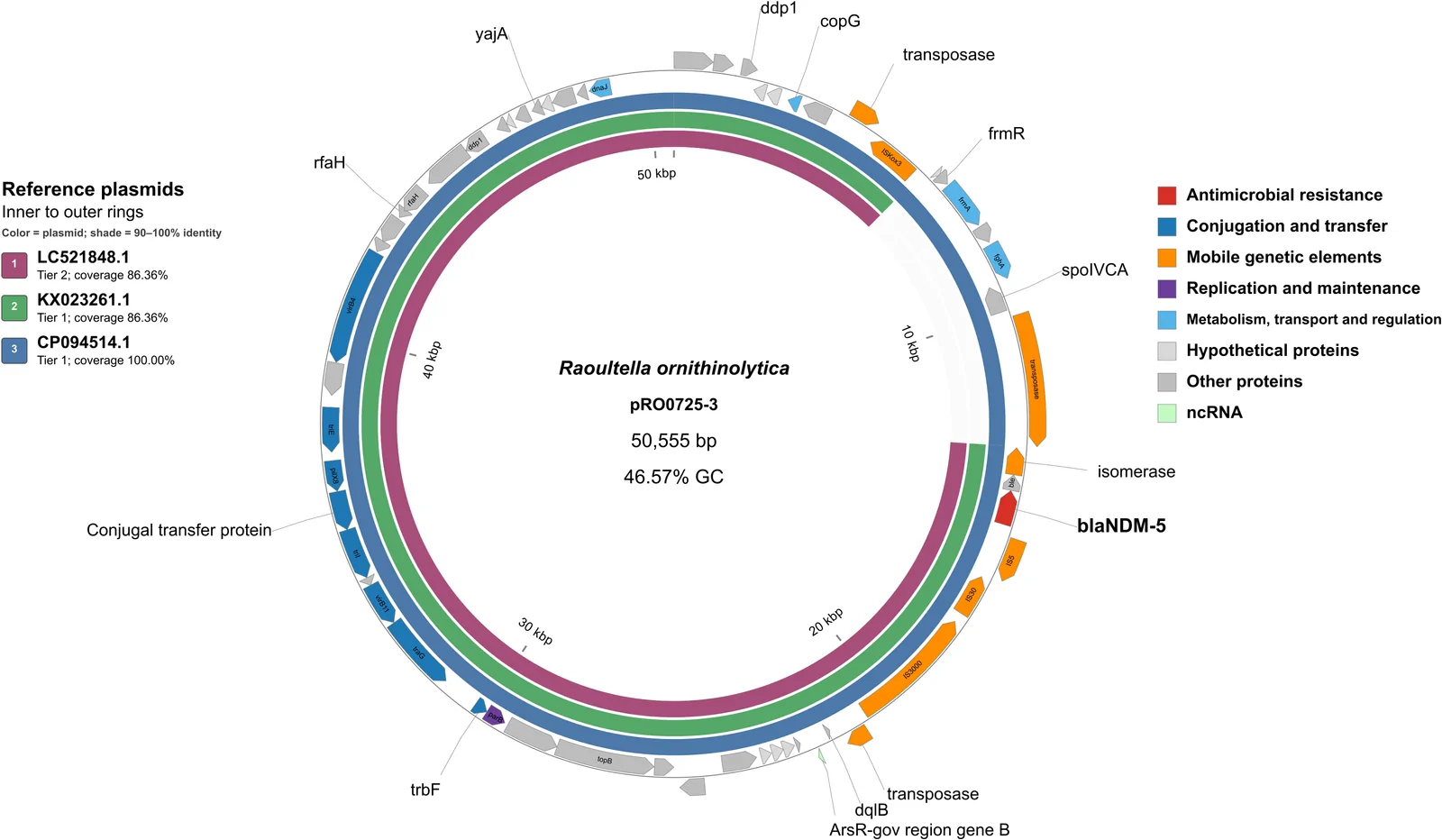
Circular comparative map of pRO0725–3 and three reference plasmids. Colored inner rings indicate regions with 90–100% nucleotide identity, and outer arrows show functionally classified genes, including *bla_NDM-5_*.

### 3.6 Genetic contexts and transferability of key resistance determinants

The truncated *mcr-10*-like element on pRO0725–1 was embedded in a mobile genetic region containing integrase, recombinase, *tnpA*, and ISEc27. A *repA–tap–copB* replication-control module was located downstream. The surrounding backbone was highly homologous to several reference plasmids carrying full-length *mcr-10.1*, although the gene was truncated in RO0725 (**Figure 7A**).

**Figure 7:**
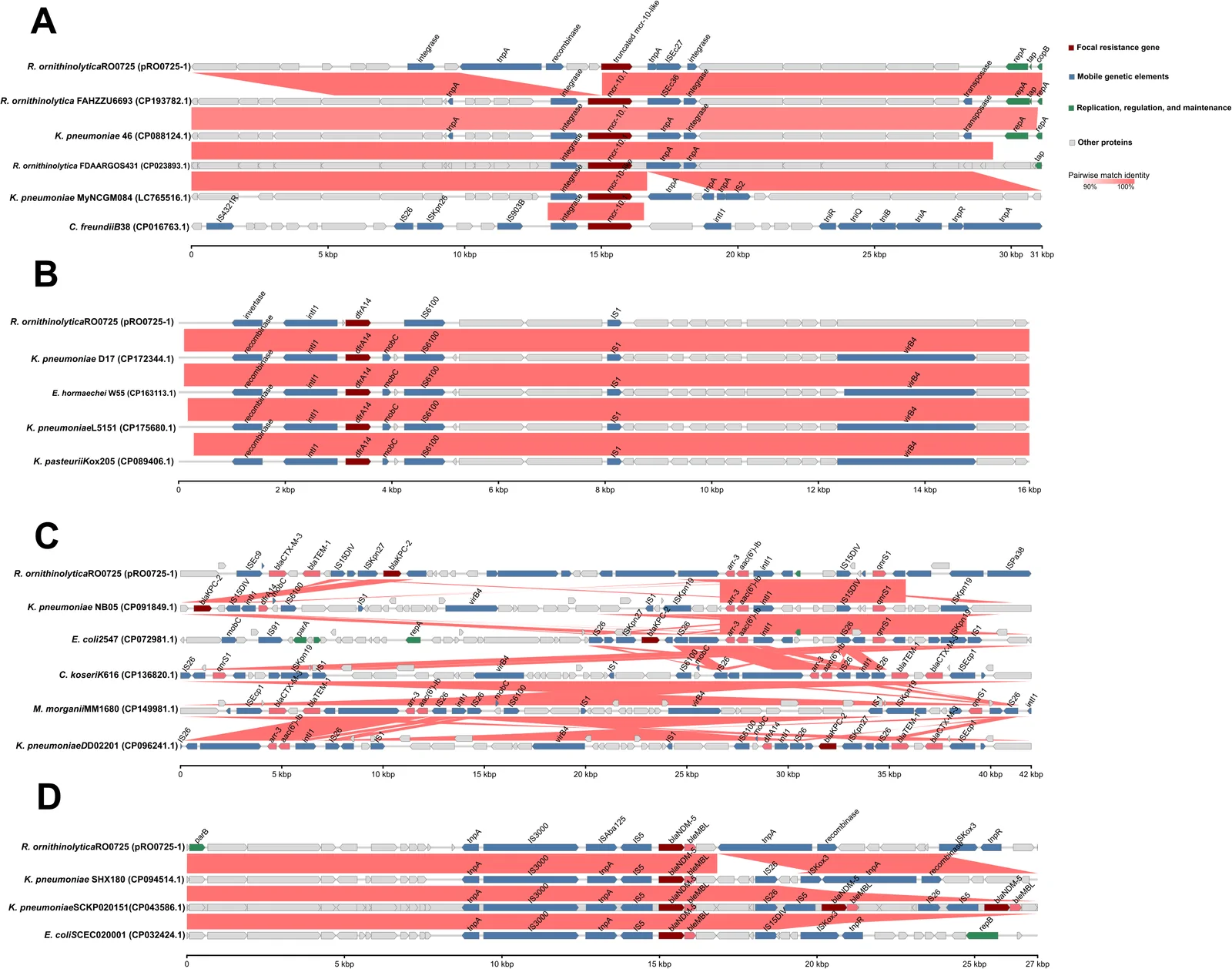
Comparative genetic contexts of key resistance regions in RO0725. **(A–D)** show the truncated *mcr-10*-like, *dfrA14*, multidrug-resistance, and *blaNDM-5* regions, respectively. Red shading indicates 90–100% nucleotide identity between sequences.

On pRO0725-2, *dfrA14* occurred in a class 1 integron-associated structure arranged as *intI1–dfrA14–IS6100–IS1*. The structure was adjacent to recombinase, *mobC*, and *virB4*. This region was highly conserved among Enterobacterales plasmids (**Figure 7B**). The approximately 42-kb multidrug-resistance region comprised ISEc9–*bla*_CTX-M-3_, *bla*_TEM-1_, ISKpn27–*bla*_KPC-2_, *arr-3–aac(6′)-Ib–intI1*, and *qnrS1* modules. It was enriched in IS15DIV, ISPa38, and other mobile elements. Reference plasmids showed extensive rearrangements and modular recombination (**Figure 7C**).

On pRO0725-3, *bla*_NDM-5_ was adjacent to *ble-*MBL in a region containing IS3000, ISAba125, IS5, and transposase-associated elements. The core structure was highly conserved among IncX3-like reference plasmids. Variation was mainly confined to downstream regions associated with IS26, ISKox3, and recombinases (**Figure 7D**).

### 3.7 Host-adaptation and virulence-associated phenotypes

After 4 h of exposure to 50 mg/mL bile salts, RO0725 had a survival rate of 18.80% ± 0.56%. This rate exceeded that of the negative control W3110 (4.10% ± 1.40%, *P* < 0.0001). It also exceeded that of *R. ornithinolytica* ATCC 31898 (14.47% ± 0.84%, *P* = 0.0043). However, it was lower than that of the positive control PBIO1289 (37.67% ± 1.19%, *P* < 0.0001) (**Figure 8A**). In the crystal-violet assay, RO0725 had an OD_570_ of 0.355 ± 0.035. This value was significantly higher than those of ATCC 31898 and the blank control but markedly lower than that of hypervirulent *K. pneumoniae* NTUH-K2044. According to the Stepanović criteria, RO0725 was classified as a weak biofilm producer (**Figure 8B**).

**Figure 8:**
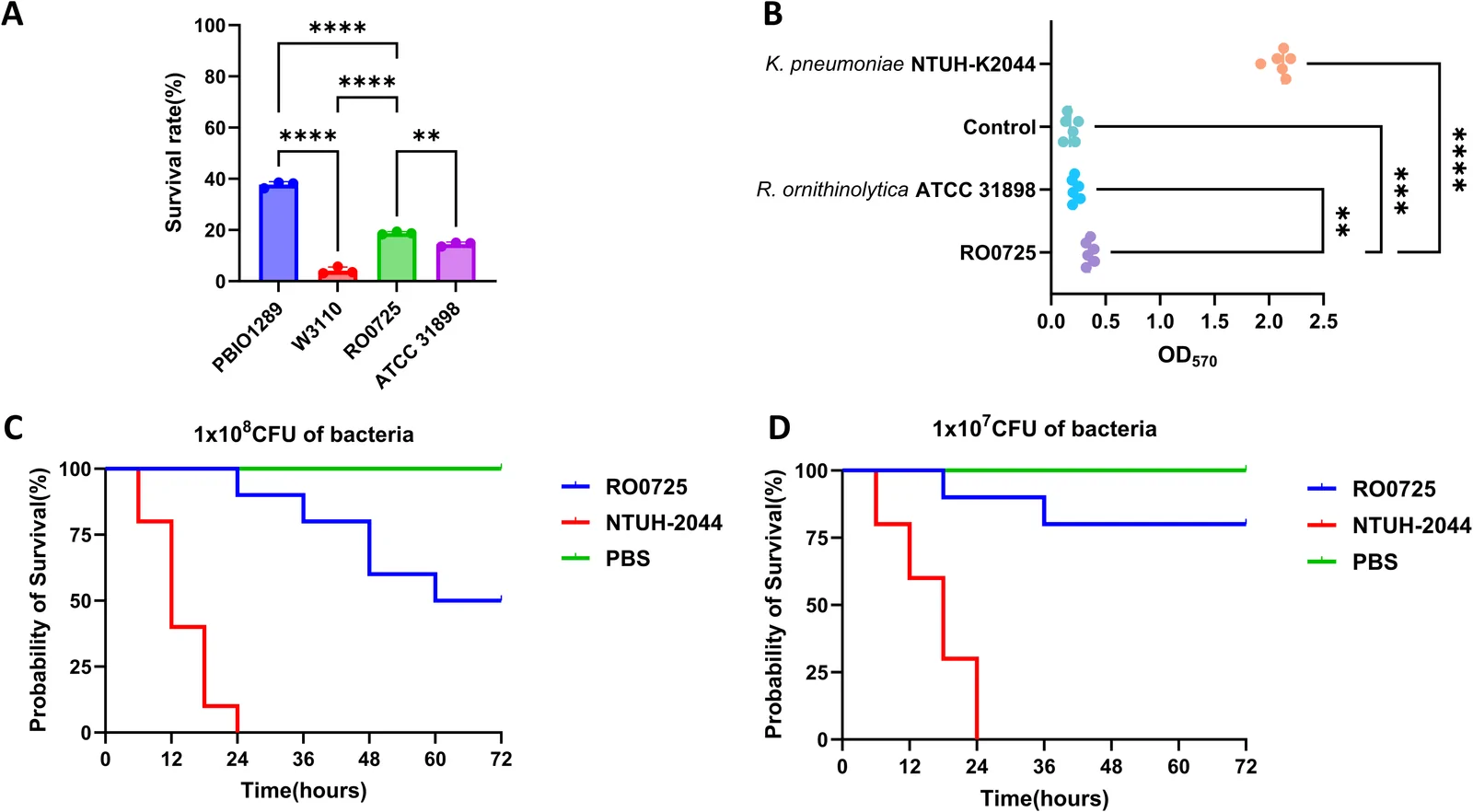
Phenotypic and transferability characterization of RO0725. **(A)** Bile-salt survival, with PBIO1289 and W3110 as positive and negative controls, respectively; **(B)** biofilm formation; **(C, D)** Galleria mellonella survival after infection with 1 × 10^8^ and 1 × 10^7^ CFU, respectively. *P < 0.05, **P < 0.01, ***P < 0.001, and ****P < 0.0001.

In the *G. mellonella* model, the 72-h survival rates after infection with 1 × 10^8^ and 1 × 10^7^ CFU of RO0725 were 50% and 80%, respectively. At the corresponding inocula, NTUH-K2044 caused 100% mortality within 24 h. No deaths occurred in the PBS group during the 72-h observation period. Survival curves differed significantly among groups at both inocula (log-rank, *P* < 0.0001) (**Figures 8C, D**).

The RO0725 genome contained several adhesion-associated genes, including *ecpA*, *fimA/C/D/E/H*, and *mrkA/B/C*. It also contained the iron-acquisition systems *ent*, *fep*, *ybt*, *irp*, *fyuA*, and *iutA*. Stress-response and outer-membrane genes included *phoP*, *rcsB*, *rpoS*, *acrAB*, and *ompA*. In addition, pRO0725–1 carried a *bcs* gene cluster associated with cellulose biosynthesis. These genetic features were consistent with the observed bile salt tolerance, biofilm formation, and moderate *in vivo* virulence. However, they do not independently establish functional causality.

## 4 Discussion

This study integrated complete genome sequencing, phylogenetic analysis, plasmid structural comparisons, conjugation assays, and host-adaptation phenotyping to characterize *R. ornithinolytica* RO0725 from postoperative drainage fluid. Its defining feature was not the acquisition of a single resistance determinant but the convergence of multiple resistance plasmids and adaptation-associated factors. Distinct plasmids carried *bla*_KPC-2_, *bla*_NDM-5_, and a truncated *mcr-10*-like element. These plasmids coexisted with determinants associated with adhesion, iron acquisition, stress tolerance, and cellulose biosynthesis. Together, these features endowed RO0725 with an extensively drug-resistant phenotype, transferable resistance plasmids, and a measurable capacity for host adaptation. They highlight the dual role of *R. ornithinolytica* as an opportunistic pathogen and a reservoir of antimicrobial-resistance determinants (Sun L. et al., 2023; Quesille-Villalobos et al., 2025). Previous reports of *bla*_KPC_, *bla*_NDM_, *mcr-10*, *tmexCD-toprJ*, and *tet(X)* in this species further illustrate its capacity to acquire mobile resistance elements (Wang CZ. et al., 2021; Sun X. et al., 2023; Jia et al., 2025; Ye et al., 2025). RO0725 was resistant to carbapenems, ceftazidime–avibactam, and aztreonam–avibactam. This finding emphasizes that treatment of isolates co-producing different carbapenemases should follow standardized antimicrobial susceptibility testing rather than rely solely on genotype.

RO0725 was closely related to environmental, food-associated, and clinical isolates from Vietnam, the United Kingdom, and Spain, respectively. However, it did not form a near-clonal cluster defined by ≤10 SNPs, providing no evidence of recent direct transmission. Its closest relatives occupied diverse ecological niches. Previous reports have also identified resistance plasmids in *R. ornithinolytica* from companion animals, river sediments, and aquaculture wastewater (Quintela-Baluja et al., 2022; Alam et al., 2023; Jia et al., 2025). Together, these findings support a possible role for this species in the circulation of resistance determinants among humans, animals, food systems, and the environment. RO0725 carried a unique combination of five plasmids. Acquired resistance determinants were distributed among multiple phylogenetic lineages rather than being restricted to a single clonal background. These patterns suggest that plasmid acquisition and recombination, rather than expansion of a single resistant clone, primarily drove its multidrug-resistance profile. However, given the single-isolate design and lack of regional surveillance data, we cannot infer the prevalence or geographic expansion of *R. ornithinolytica* in eastern China. Our findings instead suggest that multidrug resistance in this species is primarily shaped by acquisition and recombination of mobile resistance elements, with transferable IncN/IncU and IncX3-like plasmids potentially facilitating horizontal dissemination. This interpretation is consistent with long-term hospital surveillance showing repeated *bla*_KPC_ spread among species and clonal backgrounds through stable plasmid vehicles (Gomez-Simmonds et al., 2024).

Among the plasmids identified, pRO0725–2 showed the clearest dissemination potential. This IncN/IncU plasmid integrated *bla*_KPC-2_, *bla*_CTX-M-3_, *bla*_TEM-1_, *qnrS1*, and a class 1 integron-associated resistance module. It transferred to *K. pneumoniae* KP033 at a mean frequency of 6.3 × 10^-^³. Previous studies demonstrated that *bla*_KPC_-carrying IncN plasmids can disseminate across bacterial species and persist in healthcare settings (Gomez-Simmonds et al., 2022). This evidence is consistent with the conserved conjugative backbone and structurally variable resistance island observed here. The discordance between *dfrA14* and trimethoprim–sulfamethoxazole susceptibility further indicates that gene detection alone does not necessarily predict phenotypic resistance. Phenotypic resistance may also depend on gene integrity, expression level, regulatory context, and the recipient background.

Although pRO0725–3 transferred at a lower frequency, it was highly homologous to IncX3-like *bla*_NDM-5_ plasmids from diverse sources. It also retained the conserved IS3000–ISAba125–IS5–*bla*_NDM-5_–*ble*-MBL core structure. Recent clinical studies similarly documented interspecies transfer, stable maintenance, and broad dissemination of *bla*_NDM-5_ mediated by conserved IncX3 plasmids (Harada et al., 2024; Ma et al., 2024). Therefore, a relatively low transfer frequency under a single *in vitro* condition should not be interpreted as evidence of low epidemiological risk.

The *mcr-10*-like fragment carried by pRO0725–1 covered only 68.52% of the full-length reference gene. It occurred in a region enriched in integrases, recombinases, *tnpA*, and ISEc27. The plasmid backbone was closely related to those carrying intact *mcr-10.1*. This context suggests that the fragment may have arisen through mobilization followed by partial deletion or structural disruption. Full-length *mcr-10* has previously been reported with *bla*_KPC-2_ on IncF-type plasmids in environmentally derived Enterobacterales. Studies of wastewater and clinical isolates in Japan also showed that *mcr-10* may disseminate with tellurite-resistance-associated *ter* loci (Li et al., 2021; Yu et al., 2025). Nevertheless, colistin resistance in RO0725 cannot be attributed solely to the truncated element. Alterations in *phoPQ*, *pmrAB*, *mgrB*, or other lipid A modification pathways may also contribute. Although pRO0725–1 contained a complete predicted conjugative apparatus, no corresponding transconjugant was recovered. This result may reflect recipient-range restrictions, insufficient expression of the conjugation system, plasmid-associated fitness costs, or the selection conditions. It does not establish that the plasmid is intrinsically non-transferable. Beyond antimicrobial-resistance determinants, pRO0725–1 also carried a plasmid-associated cellulose biosynthesis locus. Plasmid-associated bcs loci have been reported in several Enterobacterales species, although their distribution and biological significance remain incompletely characterized. Previous examples include bcs-bearing plasmids from *Klebsiella variicola*, *Citrobacter portucalensis*, and *Escherichia coli* (Rodríguez-Medina et al., 2020; Begmatov et al., 2026; Hosseinpourlamardi et al., 2026). In RO0725, the pRO0725-1-associated locus consisted of a bcsOQABCDZ-related region with oppositely oriented bcsFG, while bcsE and bcsR could not be confirmed (**Supplementary Table S2**). The presence of an IS1N-associated element upstream and an IS6-family transposase downstream suggests a possible history of mobile-element-associated acquisition or rearrangement; however, the mechanism of acquisition remains unresolved. The coexistence of this locus with the truncated *mcr-10*-like element and other adaptation-associated genes highlights the potential accumulation of resistance-associated and persistence-related traits within a single plasmid background. Nevertheless, functional experiments are required to determine whether this bcs locus contributes to cellulose production, biofilm formation, or host adaptation.

Phenotypic analyses indicated that RO0725 was not a typical hypervirulent strain. It exhibited substantial bile salt tolerance, weak biofilm formation, and intermediate lethality in the *Galleria mellonella* model. Its acute virulence was markedly lower than that of hypervirulent *K. pneumoniae* NTUH-K2044. The *fim*, *mrk*, and *ecp* genes provide a potential genetic basis for adhesion. The ybt and iutA genes may support iron acquisition, whereas acrAB, ompA, and bcs may contribute to stress tolerance and environmental persistence. However, the functional contributions of these genes were not experimentally established. RO0725 may therefore possess sufficient host adaptation for persistence in postoperative drainage systems, implanted materials, or intensive-care environments without causing an acutely lethal phenotype*. P*revious reports have described postoperative, biliary, pulmonary, and other invasive infections caused by *R. ornithinolytica* (Prada-Avella et al., 2021; Goodman et al., 2022; Baek et al., 2023; Etani et al., 2023; De Coster et al., 2024; Huang et al., 2024; Sun et al., 2024). Accordingly, RO0725 may represent an opportunistic pathotype characterized by moderate virulence, extensive antimicrobial resistance, and transferable plasmids. Its clinical risk may arise primarily from persistent colonization, treatment failure, and transfer of resistance determinants to other *Enterobacterales* rather than exceptionally high acute virulence.

This study has several limitations. Only one clinical isolate was analyzed, and no contemporaneous patient-screening or environmental isolates were available. Conjugation experiments used KP033 as the sole recipient. The resulting transconjugants were not subjected to whole-genome sequencing. In addition, neither the truncated *mcr-10*-like element nor the putative host-adaptation genes were validated through expression or genetic studies. Despite these limitations, our findings demonstrate that *R. ornithinolytica* can serve as a convergence platform for distinct resistance plasmids and host-adaptation determinants. These results support its inclusion in hospital and One Health antimicrobial-resistance surveillance, particularly for the interspecies dissemination of IncN/IncU and IncX3-like plasmids.

## 5 Conclusion

This study showed that the postoperative *R. ornithinolytica* isolate RO0725 harbored an IncN/IncU plasmid carrying *bla*_KPC-2_ and an IncX3-like plasmid carrying *bla*_NDM-5_. It also carried a truncated *mcr-10*-like element. RO0725 exhibited experimentally confirmed transferability of resistance plasmids and measurable host-adaptation phenotypes. Its major risks are extensive antimicrobial resistance, persistent colonization, and potential dissemination of resistance genes to other *Enterobacterales*. These findings support enhanced clinical and One Health surveillance of this emerging opportunistic pathogen.

## Data Availability

The datasets presented in this study can be found in online repositories. The names of the repository/repositories and accession number(s) can be found below: https://www.ncbi.nlm.nih.gov/, GCA_059772045.1.
