# Supplementary material for: Genomic convergence of mobilome-associated multidrug resistance and host-adaptation determinants in a postoperative Raoultella ornithinolytica isolate carrying blaKPC-2, blaNDM-5, and a truncated mcr-10-like element
Source: Front Cell Infect Microbiol. 2026 Sep 25;16:1966531. doi: 10.3389/fcimb.2026.1966531 (PMC13649461; doi:10.3389/fcimb.2026.1966531)
Supplement: Supplementary file 1 [file Image1.pdf]

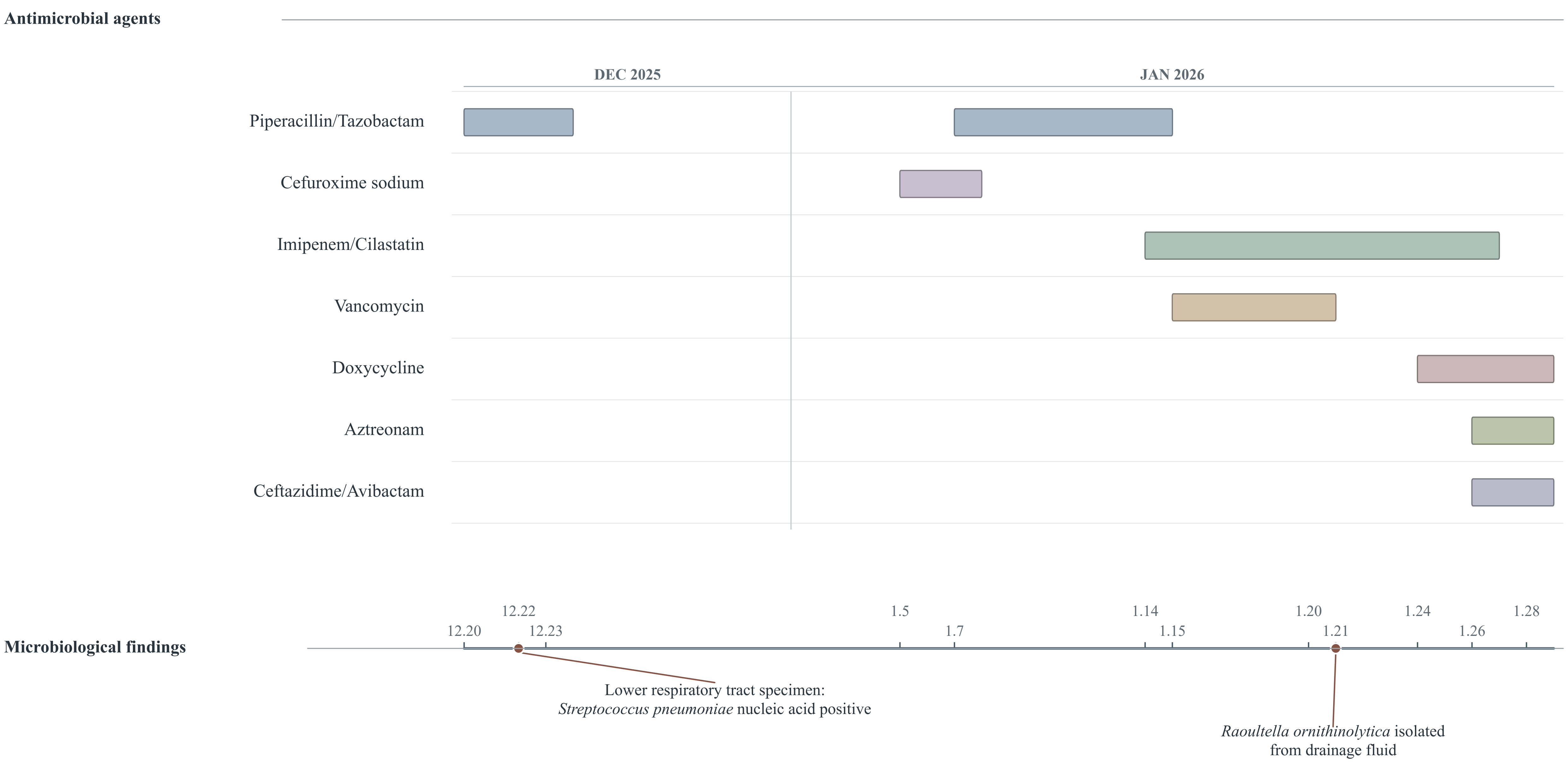

Antimicrobial agents
DEC 2025
JAN 2026
Piperacillin/Tazobactam
Cefuroxime sodium
Imipenem/Cilastatin
Vancomycin
Doxycycline
Aztreonam
Ceftazidime/Avibactam
Microbiological findings
12.20
12.22
12.23
1.5
1.7
1.14
1.15
1.20
1.21
1.24
1.26
1.28
Lower respiratory tract specimen:
Streptococcus pneumoniae nucleic acid positive
Raoultella ornithinolytica isolated
from drainage fluid
