# Supplementary material for: Genomic convergence of mobilome-associated multidrug resistance and host-adaptation determinants in a postoperative Raoultella ornithinolytica isolate carrying blaKPC-2, blaNDM-5, and a truncated mcr-10-like element
Source: Front Cell Infect Microbiol. 2026 Sep 25;16:1966531. doi: 10.3389/fcimb.2026.1966531 (PMC13649461; doi:10.3389/fcimb.2026.1966531)
Supplement: Supplementary file 2 [file Image2.pdf]

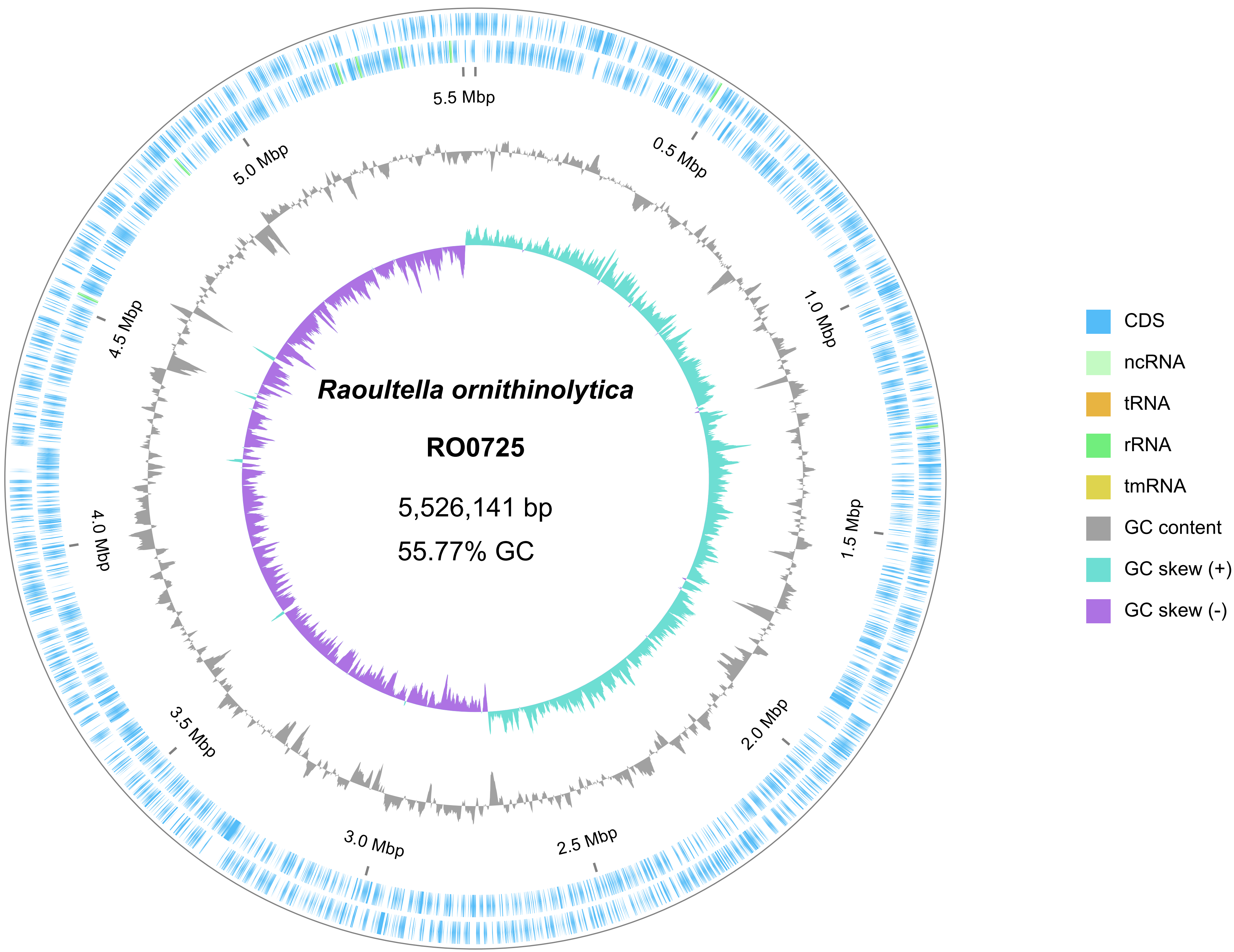
Raoultella ornithinolytica
RO0725
5,526,141 bp
55.77% GC
0.5 Mbp
1.0 Mbp
1.5 Mbp
2.0 Mbp
2.5 Mbp
3.0 Mbp
3.5 Mbp
4.0 Mbp
4.5 Mbp
5.0 Mbp
5.5 Mbp
CDS
ncRNA
tRNA
rRNA
tmRNA
GC content
GC skew (+)
GC skew (-)
