# Supplementary material for: Genomic convergence of mobilome-associated multidrug resistance and host-adaptation determinants in a postoperative Raoultella ornithinolytica isolate carrying blaKPC-2, blaNDM-5, and a truncated mcr-10-like element
Source: Front Cell Infect Microbiol. 2026 Sep 25;16:1966531. doi: 10.3389/fcimb.2026.1966531 (PMC13649461; doi:10.3389/fcimb.2026.1966531)
Supplement: Supplementary file 3 [file Image5.pdf]

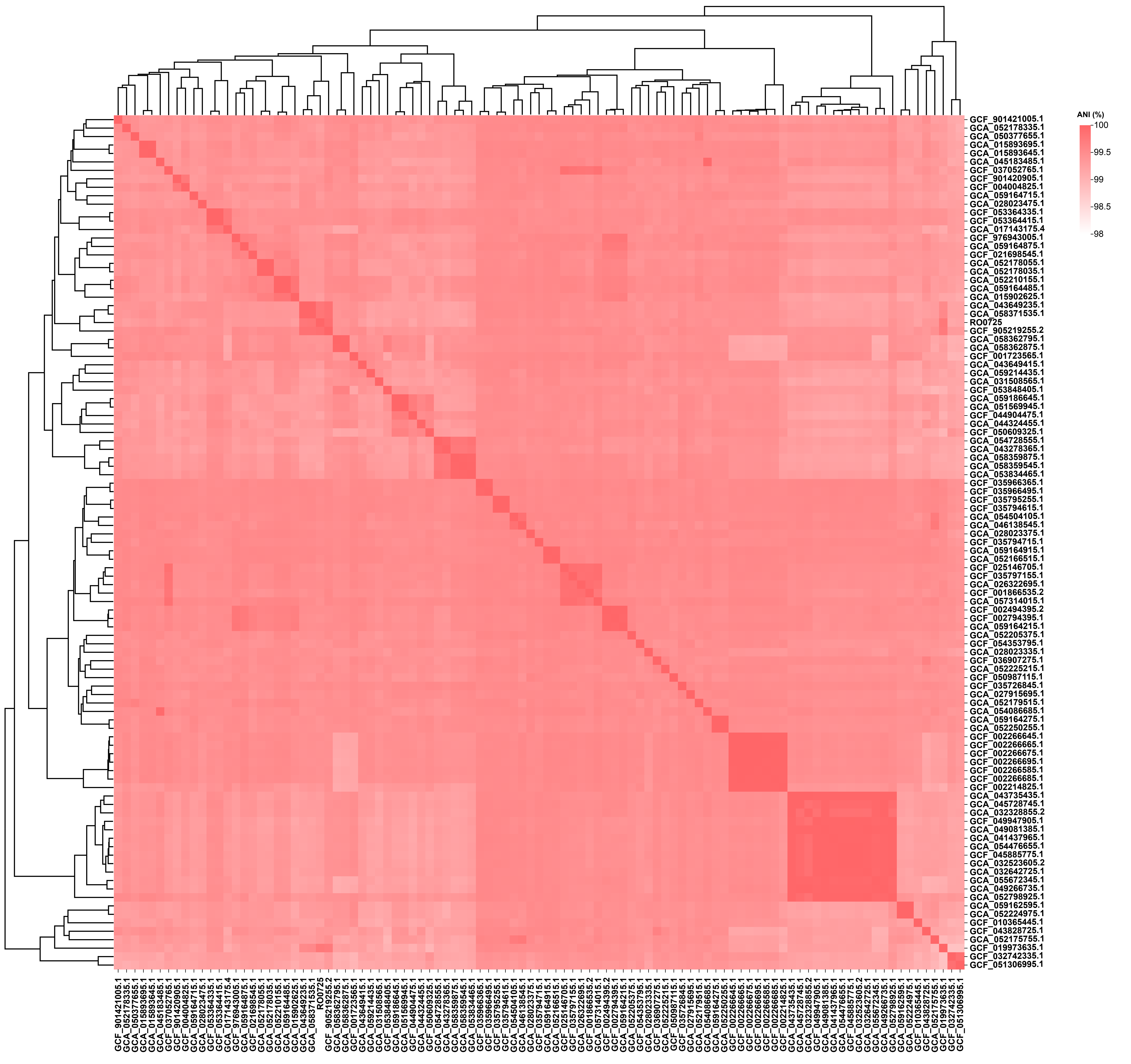
ANI (%)
100
99.5
99
98.5
98
GCF_901421005.1
GCA_052178335.1
GCA_050377655.1
GCA_015893695.1
GCA_015893645.1
GCA_045183485.1
GCF_037052765.1
GCF_901420905.1
GCF_004004825.1
GCA_059164715.1
GCA_028023475.1
GCF_053364335.1
GCF_053364415.1
GCA_017143175.4
GCF_976943005.1
GCA_059164875.1
GCF_021698545.1
GCA_052178055.1
GCA_052178035.1
GCA_052210155.1
GCA_059164485.1
GCA_015902625.1
GCA_043649235.1
GCA_058371535.1
RO0725
GCF_905219255.2
GCA_058362795.1
GCA_058362875.1
GCF_001723565.1
GCA_043649415.1
GCA_059214435.1
GCA_031508565.1
GCF_053848405.1
GCA_059186645.1
GCA_051569945.1
GCF_044904475.1
GCA_044324455.1
GCF_050609325.1
GCA_054728555.1
GCA_043278365.1
GCA_058359875.1
GCA_058359545.1
GCA_053834465.1
GCF_035966365.1
GCF_035966495.1
GCF_035795255.1
GCF_035794615.1
GCA_054504105.1
GCA_046138545.1
GCA_028023375.1
GCF_035794715.1
GCA_059164915.1
GCA_052166515.1
GCF_025146705.1
GCF_035797155.1
GCA_026322695.1
GCF_001866535.2
GCA_057314015.1
GCF_002494395.2
GCF_002794395.1
GCA_059164215.1
GCA_052205375.1
GCF_054353795.1
GCA_028023335.1
GCF_036907275.1
GCA_052225215.1
GCF_050987115.1
GCF_035726845.1
GCA_027915695.1
GCA_052179515.1
GCA_054086685.1
GCA_059164275.1
GCA_052250255.1
GCF_002266645.1
GCF_002266665.1
GCF_002266675.1
GCF_002266695.1
GCF_002266585.1
GCF_002266685.1
GCF_002214825.1
GCA_043735435.1
GCA_045728745.1
GCA_032328855.2
GCF_049947905.1
GCA_049081385.1
GCA_041437965.1
GCA_054476655.1
GCF_045885775.1
GCA_032523605.2
GCA_032642725.1
GCA_055672345.1
GCA_049266735.1
GCA_052798925.1
GCA_059162595.1
GCA_052224975.1
GCF_010365445.1
GCF_043828725.1
GCA_052175755.1
GCF_019973635.1
GCF_032742335.1
GCF_051306995.1
