# Supplementary material for: Genomic convergence of mobilome-associated multidrug resistance and host-adaptation determinants in a postoperative Raoultella ornithinolytica isolate carrying blaKPC-2, blaNDM-5, and a truncated mcr-10-like element
Source: Front Cell Infect Microbiol. 2026 Sep 25;16:1966531. doi: 10.3389/fcimb.2026.1966531 (PMC13649461; doi:10.3389/fcimb.2026.1966531)
Supplement: Supplementary file 6 [file Image8.pdf]

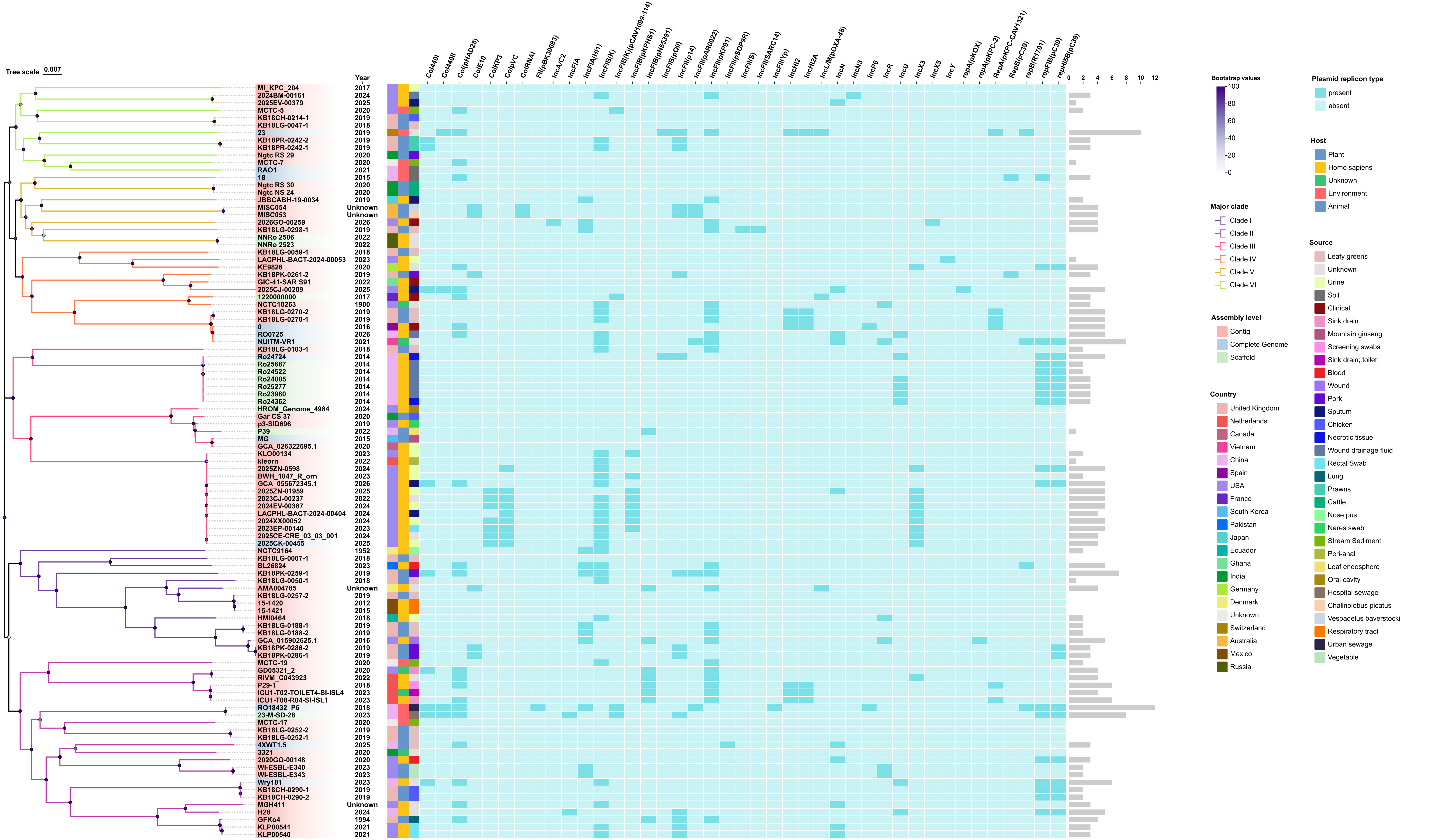

Tree scale 0.007
Year
MI_KPC_204 2017
2024BM-00161 2024
2025EV-00379 2025
MCTC-5 2020
KB18CH-0214-1 2019
KB18LG-0047-1 2018
23 2019
KB18PR-0242-2 2019
KB18PR-0242-1 2019
Ngtc RS 29 2020
MCTC-7 2020
RAO1 2021
18 2015
Ngtc RS 30 2020
Ngtc NS 24 2020
JBBCABH-19-0034 2019
MISC054 Unknown
MISC053 Unknown
2026GO-00259 2026
KB18LG-0298-1 2019
NNRo 2506 2022
NNRo 2523 2022
KB18LG-0059-1 2018
LACPHL-BACT-2024-00053 2023
KE9826 2020
KB18PK-0261-2 2019
GIC-41-SAR S91 2022
2025CJ-00209 2025
1220000000 2017
NCTC10263 1900
KB18LG-0270-2 2019
KB18LG-0270-1 2019
0 2016
RO0725 2026
NUITM-VR1 2021
KB18LG-0103-1 2018
Ro24724 2014
Ro25687 2014
Ro24522 2014
Ro24005 2014
Ro25277 2014
Ro23980 2014
Ro24362 2014
HROM_Genome_4984 2024
Gar CS 37 2020
p3-SID696 2019
P39 2022
MG 2015
GCA_026322695.1 2020
KLO00134 2023
kleorn 2022
2025ZN-0598 2024
BWH_1047_R_orn 2023
GCA_055672345.1 2026
2025ZN-01959 2025
2023CJ-00237 2022
2024EV-00387 2024
LACPHL-BACT-2024-00404 2024
2024XX00052 2024
2023EP-00140 2023
2025CE-CRE_03_03_001 2024
2025CK-00455 2025
NCTC9164 1952
KB18LG-0007-1 2018
BL26824 2023
KB18PK-0259-1 2019
KB18LG-0050-1 2018
AMA004785 Unknown
KB18LG-0257-2 2019
15-1420 2012
15-1421 2015
HMI0464 2018
KB18LG-0188-1 2019
KB18LG-0188-2 2019
GCA_015902625.1 2016
KB18PK-0286-2 2019
KB18PK-0286-1 2019
MCTC-19 2020
GD05321_2 2020
RIVM_C043923 2022
P29-1 2018
ICU1-T02-TOILET4-SI-ISL4 2023
ICU1-T08-R04-SI-ISL1 2023
RO18432_P6 2018
23-M-SD-28 2023
MCTC-17 2020
KB18LG-0252-2 2019
KB18LG-0252-1 2019
4XWT1.5 2025
3321 2020
2020GO-00148 2020
WI-ESBL-E340 2023
WI-ESBL-E343 2023
Wry181 2023
KB18CH-0290-1 2019
KB18CH-0290-2 2019
MGH411 Unknown
H28 2024
GFKo4 1994
KLP00541 2021
KLP00540 2021
Col440I
Col440II
Col(pHAD28)
ColE10
ColKP3
ColpVC
ColRNAI
FII(pBK30683)
IncA/C2
IncFIA
IncFIA(HI1)
IncFIB(K)
IncFIB(K)(pCAV1099-114)
IncFIB(pKPHS1)
IncFIB(pN55391)
IncFIB(pQil)
IncFII(p14)
IncFII(pAR0022)
IncFII(pKP91)
IncFII(pSDP9R)
IncFII(S)
IncFII(SARC14)
IncFII(Yp)
IncHI2
IncHI2A
IncL/M(pOXA-48)
IncN
IncN3
IncP6
IncR
IncU
IncX3
IncX5
IncY
repA(pKOX)
repA(pKPC-2)
RepA(pKPC-CAV1321)
RepB(pC39)
repB(R1701)
repFIB(pC39)
repHI5B(pC39)
0 2 4 6 8 10 12
Bootstrap values
100
80
60
40
20
0
Major clade
Clade I
Clade II
Clade III
Clade IV
Clade V
Clade VI
Assembly level
Contig
Complete Genome
Scaffold
Country
United Kingdom
Netherlands
Canada
Vietnam
China
Spain
USA
France
South Korea
Pakistan
Japan
Ecuador
Ghana
India
Germany
Denmark
Unknown
Switzerland
Australia
Mexico
Russia
Plasmid replicon type
present
absent
Host
Plant
Homo sapiens
Unknown
Environment
Animal
Source
Leafy greens
Unknown
Urine
Soil
Clinical
Sink drain
Mountain ginseng
Screening swabs
Sink drain; toilet
Blood
Wound
Pork
Sputum
Chicken
Necrotic tissue
Wound drainage fluid
Rectal Swab
Lung
Prawns
Cattle
Nose pus
Nares swab
Stream Sediment
Peri-anal
Leaf endosphere
Oral cavity
Hospital sewage
Chalinolobus picatus
Vespadelus baverstocki
Respiratory tract
Urban sewage
Vegetable
