# Supplementary material for: Genomic convergence of mobilome-associated multidrug resistance and host-adaptation determinants in a postoperative Raoultella ornithinolytica isolate carrying blaKPC-2, blaNDM-5, and a truncated mcr-10-like element
Source: Front Cell Infect Microbiol. 2026 Sep 25;16:1966531. doi: 10.3389/fcimb.2026.1966531 (PMC13649461; doi:10.3389/fcimb.2026.1966531)
Supplement: Supplementary file 7 [file Image9.pdf]

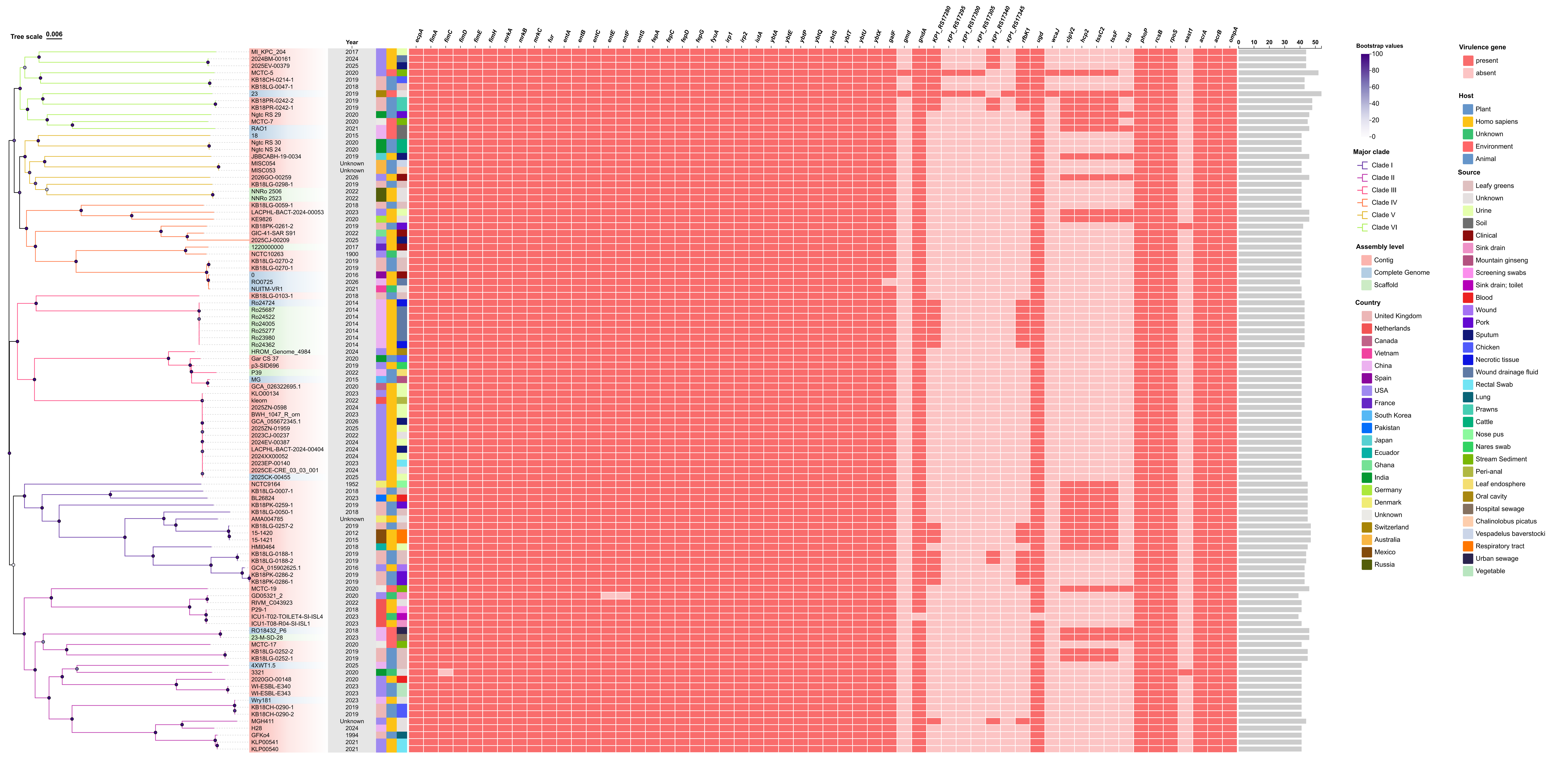

Tree scale 0.006
Year
ecpA fimA fimC fimD fimE fimH mrkA mrkB mrkC fur entA entB entC entE entF entS fepA fepC fepD fepG fyuA irp1 irp2 iutA ybtA ybtE ybtP ybtQ ybtS ybtT ybtU ybtX galF gmd gndA KP1_RS17280 KP1_RS17285 KP1_RS17300 KP1_RS17305 KP1_RS17340 KP1_RS17345 rfbK1 ugd wcaJ clpV2 hcp2 tssC2 tssF tssI phoP rcsB rpoS east1 acrA acrB ompA
0 10 20 30 40 50
Bootstrap values
100
80
60
40
20
0
Major clade
Clade I
Clade II
Clade III
Clade IV
Clade V
Clade VI
Assembly level
Contig
Complete Genome
Scaffold
Country
United Kingdom
Netherlands
Canada
Vietnam
China
Spain
USA
France
South Korea
Pakistan
Japan
Ecuador
Ghana
India
Germany
Denmark
Unknown
Switzerland
Australia
Mexico
Russia
Virulence gene
present
absent
Host
Plant
Homo sapiens
Unknown
Environment
Animal
Source
Leafy greens
Unknown
Urine
Soil
Clinical
Sink drain
Mountain ginseng
Screening swabs
Sink drain; toilet
Blood
Wound
Pork
Sputum
Chicken
Necrotic tissue
Wound drainage fluid
Rectal Swab
Lung
Prawns
Cattle
Nose pus
Nares swab
Stream Sediment
Peri-anal
Leaf endosphere
Oral cavity
Hospital sewage
Chalinolobus picatus
Vespadelus baverstocki
Respiratory tract
Urban sewage
Vegetable
