# Supplementary material for: Genomic convergence of mobilome-associated multidrug resistance and host-adaptation determinants in a postoperative Raoultella ornithinolytica isolate carrying blaKPC-2, blaNDM-5, and a truncated mcr-10-like element
Source: Front Cell Infect Microbiol. 2026 Sep 25;16:1966531. doi: 10.3389/fcimb.2026.1966531 (PMC13649461; doi:10.3389/fcimb.2026.1966531)
Supplement: Supplementary file 8 [file Table1.docx]

Table S1 Primers and their sequences used for PCR

| mcr10-5F | ACTGGCCGGTGTTGCTGCATTT |
| --- | --- |
| mcr10-5R | TCAGCGAAGCGAGCATGGAAAGC |
| NDM5-F | GGTTTGGCGATCTGGTTTTC |
| NDM5-R | CGGAATGGCTCATCACGATC |
| KPC-2F | GCGGCTCCATCGGTGTGTA |
| KPC-2R | TGGCGGCGGCGTTATCA |
