# Supplementary material for: Genomic convergence of mobilome-associated multidrug resistance and host-adaptation determinants in a postoperative Raoultella ornithinolytica isolate carrying blaKPC-2, blaNDM-5, and a truncated mcr-10-like element
Source: Front Cell Infect Microbiol. 2026 Sep 25;16:1966531. doi: 10.3389/fcimb.2026.1966531 (PMC13649461; doi:10.3389/fcimb.2026.1966531)
Supplement: Supplementary file 9 [file Table2.docx]

Table S2A. Comparison of plasmids carrying cellulose biosynthesis-associated genes

| **Plasmid** | **bcs genes detected** | **ARGs carried** | **Other adaptive genes** | **Mobile genetic elements** |
| --- | --- | --- | --- | --- |
| pRO0725-1 (pRO0725-1) | bcsA, bcsB, bcsC, bcsD, bcsF, bcsG, bcsO, bcsQ, bcsZ | Putative truncated mcr-10-like element (prior project; function not demonstrated); No hit in existing screen; not proof of absence | arsR, dps, czcD, terY, arsH, arsA, arsC, arsB, arsD | IS1 family IS1N transposase ORF A [421 bp]; IS1 family transposase ORF B [720 bp]; IS6 family transposase [1395 bp] |
| pFAHZZU6693-KPC-MCR (CP193782.1) | bcsA, bcsB, bcsC, bcsD, bcsF, bcsG, bcsO, bcsQ [pseudo], bcsZ | blaKPC-2, mcr-10.1 | arsH, arsA, arsC, arsB, arsD, arsR | IS110-like element IS4321 family transposase [381 bp]; IS1-like element IS1N family transposase [421 bp]; recombinase family protein [2485 bp] |
| pNUITM-VR1_2 (AP025011.1) | bcsA, bcsB, bcsC, bcsD, bcsF, bcsG, bcsO, bcsQ, bcsZ | mcr-10.1, qnrS1, aph(6)-Id, aph(3'')-Ib, toprJ1, tmexD1, tmexC2 | arsH_2, arsD_1, arsA_1, arsC, arsB_2, arsA_2, arsD_2, arsR | IS110 family transposase [381 bp]; IS6 family transposase [2285 bp]; transposase [4027 bp] |
| pCYTER (PQ136552.1) | bcsA, bcsB, bcsC, bcsD, bcsF, bcsG, bcsO, bcsQ, bcsZ | No hit in existing screen; not proof of absence | Not determined | transposase [381 bp]; transposase [2273 bp]; transposase [4884 bp] |
| pKOS1-1-2 (CP199073.1) | bcsA, bcsB, bcsC, bcsD, bcsE [pseudo], bcsF, bcsG, bcsO, bcsQ [pseudo], bcsZ | No ARGs reported on this plasmid [Begmatov et al. 2026]; no new uniform screen | arsH, arsC | IS6-like element IS26 family transposase [63 bp]; IS5-like element ISEc68 family transposase [210 bp]; IS5 family transposase [2617 bp] |
| pKv8917 (CP063404.1) | bcsA, bcsB, bcsC | Not determined | terE, terD, terC, terB, terA | transposase [326 bp]; IS6 family transposase [640 bp]; IS110 family transposase [1307 bp] |
| unnamed1 (CP061765.1) | bcsA, bcsB, bcsC, bcsD, bcsE [pseudo], bcsF, bcsG, bcsO, bcsQ, bcsZ | Not determined | arsC, arsB, arsA, arsD, arsR | IS1 family transposase [15 bp]; IS5-like element ISEc68 family transposase [210 bp]; Tn3 family transposase [796 bp] |
| A (CP010243.1) | bcsABC (reported EcType 2); bcsA and cellulose synthase annotations; divergent from RO0725 | Not determined | Not determined | Not determined |

Table S2B. Detailed comparison of bcs operon organization

| **Plasmid** | **bcsA** | **bcsB** | **bcsC** | **bcsD** | **Other bcs-associated genes** | **Operon organization** | **Genetic context** | **Similarity to RO0725** |
| --- | --- | --- | --- | --- | --- | --- | --- | --- |
| pRO0725-1 (pRO0725-1) | Detected | Detected | Detected/homology-supported | Detected/homology-supported | bcsO, bcsQ, bcsZ, bcsG, bcsF | bcsO+; bcsQ+; bcsA+; bcsB+; bcsC+; bcsD+; bcsZ+; bcsG-; bcsF- | IS1 family IS1N transposase ORF A [421 bp]; IS1 family transposase ORF B [720 bp]; IS6 family transposase [1395 bp] | Coverage 100.0%; HSP-weighted identity 100.0%; local 13,354-bp locus, not whole-plasmid ANI |
| pFAHZZU6693-KPC-MCR (CP193782.1) | Detected | Detected | Detected/homology-supported | Detected/homology-supported | bcsO, bcsQ [pseudo], bcsZ, bcsG, bcsF | bcsO+; bcsQ+; bcsA+; bcsB+; bcsC+; bcsD+; bcsZ+; bcsG-; bcsF- | IS110-like element IS4321 family transposase [381 bp]; IS1-like element IS1N family transposase [421 bp]; recombinase family protein [2485 bp] | Coverage 100.0%; HSP-weighted identity 99.985%; local 13,354-bp locus, not whole-plasmid ANI |
| pNUITM-VR1_2 (AP025011.1) | Detected | Detected | Detected/homology-supported | Detected/homology-supported | bcsO, bcsQ, bcsZ, bcsG, bcsF | bcsO+; bcsQ+; bcsA+; bcsB+; bcsC+; bcsD+; bcsZ+; bcsG-; bcsF- | IS110 family transposase [381 bp]; IS6 family transposase [2285 bp]; transposase [4027 bp] | Coverage 100.0%; HSP-weighted identity 99.985%; local 13,354-bp locus, not whole-plasmid ANI |
| pCYTER (PQ136552.1) | Detected | Detected | Detected/homology-supported | Detected/homology-supported | bcsO, bcsQ, bcsZ, bcsG, bcsF | bcsO+; bcsQ+; bcsA+; bcsB+; bcsC+; bcsD+; bcsZ+; bcsG-; bcsF- | transposase [381 bp]; transposase [2273 bp]; transposase [4884 bp] | Coverage 100.0%; HSP-weighted identity 99.993%; local 13,354-bp locus, not whole-plasmid ANI |
| pKOS1-1-2 (CP199073.1) | Detected | Detected | Detected/homology-supported | Detected/homology-supported | bcsQ [pseudo], bcsG, bcsF, bcsZ, bcsO, bcsE [pseudo] | bcsO+; bcsQ+; bcsA+; bcsB+; bcsC+; bcsD+; bcsZ+; bcsG-; bcsF-; bcsE- | IS6-like element IS26 family transposase [63 bp]; IS5-like element ISEc68 family transposase [210 bp]; IS5 family transposase [2617 bp] | Coverage 97.656%; HSP-weighted identity 82.083%; local 13,354-bp locus, not whole-plasmid ANI |
| pKv8917 (CP063404.1) | Detected | Detected | Detected/homology-supported | Not determined | Not determined | bcsA+; bcsB+; bcsC+ | transposase [326 bp]; IS6 family transposase [640 bp]; IS110 family transposase [1307 bp] | Coverage 67.246%; HSP-weighted identity 91.406%; local 13,354-bp locus, not whole-plasmid ANI |
| unnamed1 (CP061765.1) | Detected | Detected | Detected/homology-supported | Detected/homology-supported | bcsQ, bcsG, bcsF, bcsZ, bcsO, bcsE [pseudo] | bcsO+; bcsQ+; bcsA+; bcsB+; bcsC+; bcsD+; bcsZ+; bcsG-; bcsF-; bcsE- | IS1 family transposase [15 bp]; IS5-like element ISEc68 family transposase [210 bp]; Tn3 family transposase [796 bp] | Coverage 97.656%; HSP-weighted identity 82.075%; local 13,354-bp locus, not whole-plasmid ANI |
| A (CP010243.1) | Reported EcType 2; divergent homologue | Reported EcType 2; divergent homologue | Reported EcType 2; divergent homologue | Not determined | Not determined | bcsABC (literature); not inferred from RO0725 nucleotide threshold | Not determined | Only 2.561% query coverage; 68.986% identity over this short match is not a meaningful locus-wide similarity |
